# Atom‐Modified gDNA Enhances Cleavage Activity of TtAgo Enabling Ultra‐Sensitive Nucleic Acid Testing

**DOI:** 10.1002/advs.202403120

**Published:** 2024-05-10

**Authors:** Jun Zhang, Miaomiao Chen, Huan Jiang, Huifang Sun, Jianing Ren, Xin Yang, Shanshan Liu, Dongsheng Wang, Zhen Huang, Jianping Liu, Daiyuan Ma, Xiaolan Guo, Guangcheng Luo

**Affiliations:** ^1^ Department of Clinical Laboratory Affiliated Hospital of North Sichuan Medical College School of Laboratory Medicine & Translational Medicine Research Center North Sichuan Medical College Nanchong 637000 China; ^2^ Key Laboratory of Bio‐Resource and Eco‐environment of Ministry of Education College of Life Sciences Sichuan University Chengdu Sichuan 610064 China; ^3^ Department of Oncology & Department of Rheumatology and Immunology Affiliated Hospital of North Sichuan Medical College Nanchong 637000 China; ^4^ Department of Clinical Laboratory Sichuan Cancer Hospital School of Medicine University of Electronic Science and Technology of China Chengdu 610041 China

**Keywords:** chemically modified gDNA, dsDNA cleavage, fluorine modification, nucleic acid testing, TtAgo

## Abstract

The DNA‐guided (gDNA) Argonaute from Thermus thermophilus (TtAgo) has little potential for nucleic acid detection and gene editing due to its poor dsDNA cleavage activity at relatively low temperature. Herein, the dsDNA cleavage activity of TtAgo is enhanced by using 2′‐fluorine (2′F)‐modified gDNA and developes a novel nucleic acid testing strategy. This study finds that the gDNA with 2′F‐nucleotides at the 3′‐end (2′F‐gDNA) can promote the assembly of the TtAgo‐guide‐target ternary complex significantly by increasing its intermolecular force to target DNA and TtAgo, thereby providing ≈40‐fold activity enhancement and decreasing minimum reaction temperature from 65 to 60 °C. Based on this outstanding advance, a novel nucleic acid testing strategy is proposed, termed FAST, which is performed by using the 2′F‐gDNA/TtAgo for target recognition and combining it with Bst DNA polymerase for nucleic acid amplification. By integrating G‐quadruplex and Thioflavin T, the FAST assay achieves one‐pot real‐time fluorescence analysis with ultra‐sensitivity, providing a limit of detection up to 5 copies (20 µL reaction mixture) for miR‐21 detection. In summary, an atom‐modification‐based strategy has been developed for enhancing the cleavage activity of TtAgo efficiently, thereby improving its practicability and establishing a TtAgo‐based nucleic acid testing technology with ultra‐sensitivity and high‐specificity.

## Graphical abstract

The primary cause for the poor dsDNA cleavage activity of TtAgo is the weak intermolecular force among gDNA TtAgo and target DNA, which impedes TtAgo‐guide‐target ternary complex formation. In this study, the atom‐modification‐based FATE strategy has been developed (the 2’‐hydrogen of guide DNA is substituted with fluorine atom) to increase the Tm and binding affinity of gDNA, thereby realizing unprecedented activity enhancement of TtAgo and improving its practical application potential significantly.

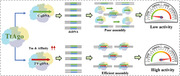

## 1 Introduction

The clustered regularly interspaced short palindromic repeat and their associated protein (CRISPR‐Cas), as a programmable nuclease, employs guide RNA (gRNA) to precisely cleave target dsDNA and has been widely used for genome editing and nucleic acid detection.^[^ ^1^, ^2^ ^]^ However, the CRISPR‐Cas system can't be applied for the dsDNA without protospacer‐adjacent motif (PAM), therefore reducing its applications.^[^ ^3^, ^4^ ^]^ Further, the susceptibility and relatively high cost of RNA also reduce its availability.

Argonautes (Ago) are a family of endonucleases that can use 5′‐phosphorylated short DNA as guide (gDNA) to cleave targets,^[^ ^5^, ^6^ ^]^ and play critical roles in gene expression and defense against foreign nucleic acid. The Ago differs from the Cas in many ways. For example, the PAM is indispensable for Cas‐mediated dsDNA cleavage, but unrequired for Ago; the Cas only exists in prokaryotes, whereas the Ago is preserved through evolution and virtually exists in all organisms; the Cas uses RNA as guide only, whereas Ago can use DNA as guide; the gRNA must have a special 5′‐end secondary structure for Cas binding, whereas no specific secondary structure is required for the gDNA. Due to the advantages of no PAM requirement, gDNA simplicity, and gDNA stability, the gDNA/Ago system provides potential for more convenient molecular diagnosis and gene editing, and thus receiving extensive attention recently.

The Ago from the thermophilic bacterium Thermus thermophilus (TtAgo), employing DNA as guide, has efficient cleavage activity for single‐strand DNA (ssDNA), but has poor activity for dsDNA cleavage, especially in circumstances with relatively low temperature (<70 °C).^[^ ^7^ ^]^ As a result, TtAgo's application for molecular diagnosis is limited.^[^ ^5^ ^]^ For example, TtAgo has little application potentials for one‐pot isothermal amplification, because almost all isothermal amplification assays work below 65 °C.^[^ ^8^, ^9^, ^10^ ^]^ Although the TtAgo has efficient dsDNA cleavage activity at 75–80 °C, this high reaction temperature makes the TtAgo‐based in vitro molecular diagnosis and in vivo gene editing almost impossible.^[^ ^11^ ^]^ Thus, enhancing dsDNA cleavage activity and reducing the reaction temperature of TtAgo are critical for broadening its application.

To explore new strategies for cleavage activity enhancement of TtAgo, it is necessary to take advantage of the structure‐function understanding. The structural studies of TtAgo ternary complexes have provided insights into the mechanism of TtAgo‐mediated target cleavage.^[^ ^12^, ^13^ ^]^ The TtAgo adopts a bilobal scaffold, which consists of N‐terminal lobe (PAZ domains and linker L1), linker L2, and C‐terminal lobe (MID and PIWI domains). First, the gDNA binds to TtAgo by anchoring its 5′‐phosphorylated end and 3′‐ hydroxyl end to the MID and PAZ domain pockets, respectively. Then, the binary complex binds to the target nucleic acid, and further form a guide‐target duplex (a helical conformation closer to the canonical A‐form). Finally, the ternary complex transforms into a cleavage‐activated conformation and executes target cleavage. These structural insights indicated that the gDNA‐TtAgo complex assembling, A‐form duplex formation, and close‐knit TtAgo‐guide‐target complex generation are critical for the TtAgo‐mediated target cleavage. Since the gDNA has identical binding affinity to the homologous strand of the target dsDNA, the TtAgo‐gDNA complex hardly invades into the target dsDNA, thereby providing poor activity for dsDNA cleavage. Enlightened by this mechanism understanding, we proposed the hypothesis that the dsDNA cleavage activity of TtAgo can be significantly enhanced by increasing the binding affinity of gDNA via chemical modification.

Previous studies reported that oligonucleotides with appropriately chemical modifications on the sugar, backbone, nucleobase, and 3′‐ and 5′‐terminal can increase their binding affinity to the complementary DNA significantly.^[^ ^14^, ^15^, ^16^ ^]^ Fluorine is highly electronegative, and 2′‐fluoro (2′F) oligonucleotides adopt C3’‐endo conformations, which provide significantly increased binding affinity and a maximum increase in melting temperature (Tm) of 2.5 °C per modified nucleotide. Locked nucleic acid (LNA), which links the 2′‐oxygen and 4′‐carbon of ribose, shows unprecedented increases in binding affinity, elevating Tm by 5–8 °C per modification.^[^ ^17^ ^]^ Further, DNA with 2′‐O‐Methyl (2′OMe),^[^ ^18^ ^]^ 2′‐O‐methoxyethyl (2′MOE)^[^ ^15^ ^]^ or minor groove binder (MGB)^[^ ^19^ ^]^ modifications also provides higher binding affinity than the corresponding natural DNA. On the basis of our hypothesis, we predicted that the above‐mentioned chemical modifications are potential candidates for enhancing TtAgo's dsDNA cleavage activity.

Thus, we investigated the potentials for chemical modifications mediated TtAgo dsDNA cleavage activity in this study. We enhanced the TtAgo's dsDNA cleavage activity by using gDNA with 2′F modification and developed a novel isothermal amplification method (2′F‐gDNA/TtAgo system assisted isothermal nucleic acid testing, FAST). The 2′F‐gDNA/TtAgo system provided approximately 40‐fold activity enhancement for dsDNA cleavage compared to the canonical counterpart and decreased minimum reaction temperature from 65 °C to 60 °C. Furthermore, the FAST assay facilitated unprecedented activity improvement, providing single‐molecule sensitivity and single‐nucleotide specificity, thereby realizing TtAgo's practical application and providing a useful tool for rapid molecular diagnosis.

## 2 Results

### 2.1 2′F Modification Facilitates TtAgo‐gDNA‐Target Ternary Complex Formation

**Figure 1:**
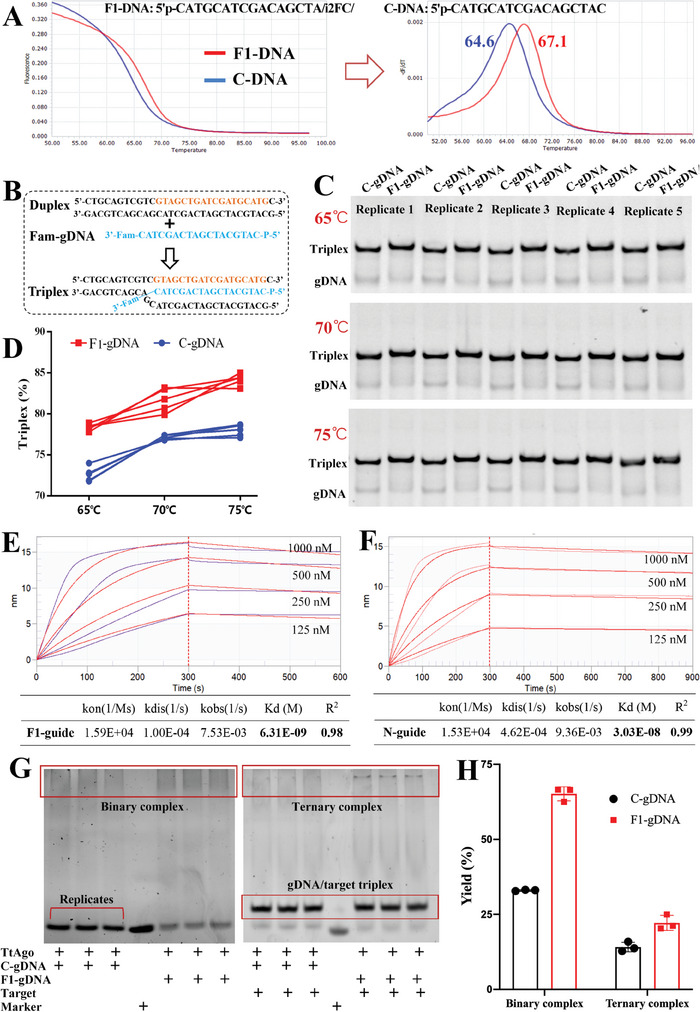
2′F‐modified gDNA facilitates TtAgo‐gDNA‐target complex formation. A) Tm comparison between F1‐gDNA and C‐gDNA. The Tm analysis based on intercalating‐dye was carried out by using unlabeled gDNA. B) The scheme for strand displacement analysis. C) Comparison of strand displacement ability between FAM‐labeled F1‐gDNA and C‐gDNA. D) Data from Figure 1C based on gray value. E) Binding affinity of F1‐gDNA. F) Binding affinity of C‐gDNA. G) Assembly efficiency analysis of gDNA/TtAgo/target complex. H) Data from Figure 1G based on grayscale. Assembly efficiency was analyzed by electrophoretic mobility shift assay (EMSA). Since the gDNA/TtAgo binary complex diffused during electrophoresis, its assembly efficiency was defined as gDNA's decrement. The assembly efficiency of the ternary complex was defined as grayscale ratio of ternary complex to marker. Comparing to C‐gDNA, the 2′F‐modified gDNA provides higher Tm and binding affinity, therefore offering enhanced strand displacement capacity and assembly efficiency.

Publications reported that an A‐form helix and a close‐knit TtAgo‐guide‐target complex are critical for the TtAgo‐mediated target cleavage.^[^ ^21^, ^22^ ^]^ RNA duplex is known to adopt an A‐form helix and exhibits higher stability than the DNA counterpart.^[^ ^23^ ^]^ Therefore, we assumed that 2′F modified gDNA may have potential to trigger better dsDNA cleavage than the canonical counterpart due to its similar structure and function to RNA. To investigate this assumption, we performed Tm analysis, biomolecular interaction analysis, and molecular docking. Data showed that the 2′F modified F1‐gDNA (single 2′F‐nucleotide at 3‐end) showed significantly higher Tm than canonical guide DNA (C‐gDNA, **Figure** **1**A). It is theoretical that the higher Tm of gDNA, the more possible to form gDNA‐target duplex. As expected, the F1‐gDNA displaced the homologous strand more efficiently than the C‐gDNA did (Figure 1B–D; Figure S2, Supporting Information) during 65–75 °C, therefore facilitating the gDNA‐dsDNA triplex formation. Further, biomolecular interaction analysis showed that the F1‐gDNA exhibited higher binding affinity to TtAgo than the corresponding C‐gDNA (Figure 1E,F). This binding affinity enhancement was supported by the data from molecular docking analysis, which showed that comparing to the corresponding C‐gDNA, the 2′F modified gDNA provides additional intermolecular contacts to the D159, R106, and W156 residues in TtAgo's PAZ domain (Figure S3, Supporting Information). Thanks to the Tm increase and binding affinity enhancement of 2′F‐gDNA, the assembly efficiency of F‐gDNA/TtAgo binary complex and F‐gDNA/TtAgo/target ternary complex were significantly elevated (Figure 1G,H). These data collectively indicated that the 2′F‐gDNA can facilitate TtAgo‐guide‐target complex formation, and offer great potential to enhance the dsDNA cleavage activity of TtAgo.

### 2.2 2′F‐gDNA Enhances dsDNA Cleavage Activity of TtAgo

**Figure 2:**
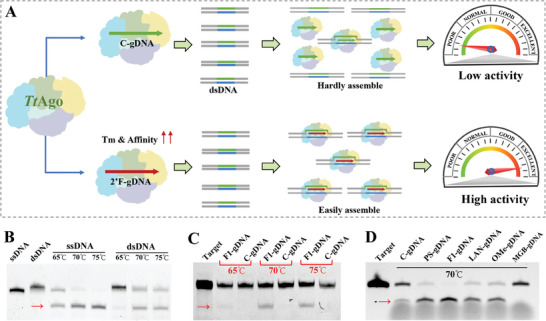
TtAgo activity enhanced by 2′F‐modified gDNA. A) The hypothesis of 2′F‐gDNA mediated dsDNA cleavage activity enhancement. The dsDNA cleavage activity of TtAgo was orchestrated by Tm and binding affinity of gDNA. B) C‐gDNA/TtAgo mediated ssDNA and dsDNA cleavage. C) Comparison of dsDNA cleavage activity between F1‐gDNA and C‐gDNA. D) TtAgo worked with 2′OMe‐, PS‐, LNA‐ or MGB‐modified gDNA. The 2′F‐modified gDNA provides the most efficient activity enhancement. The red arrows refer to the cleavage product of the target dsDNA.

Due to the capacity for facilitating TtAgo‐guide‐target complex assembly, we reasonably speculated that 2′F‐gDNA has the potential to enhance the dsDNA cleavage activity of TtAgo (**Figure** **2**A). Consistent with the previous publication,^[^ ^7^ ^]^ our data indicated that the C‐gDNA/TtAgo exhibited high cleavage activity for ssDNA, but poor cleavage activity for dsDNA (Figure 2B). In contrast, when using F1‐gDNA, the TtAgo offered significant dsDNA cleavage activity enhancement over a wide range of temperature (Figure 2C). This result is perfectly consistent with our hypothesis. According to our hypothesis, the increase of Tm and binding affinity is fundamental for dsDNA cleavage activity enhancement. It reasonably inferred that other modifications with elevated Tm and binding affinity also can improve the dsDNA cleavage activity of TtAgo. Therefore, the gDNA with 2′OMe, phosphorothioate (PS), LNA, or MGB modification was further analyzed. Data demonstrated that all the above‐mentioned modifications increased Tm of gDNA (Figure S4, Supporting Information), but not all of them can enhance dsDNA cleavage activity (Figure 2D). Although the MGB modified gDNA had the maximum Tm increase,^[^ ^19^ ^]^ it showed little activity for dsDNA cleavage. These data indicated that besides Tm increase, other factors, such as binding affinity, molecule interaction, and complex structure, are implicated in TtAgo activity.

### 2.3 The Performance of FATE Reaction

**Figure 3:**
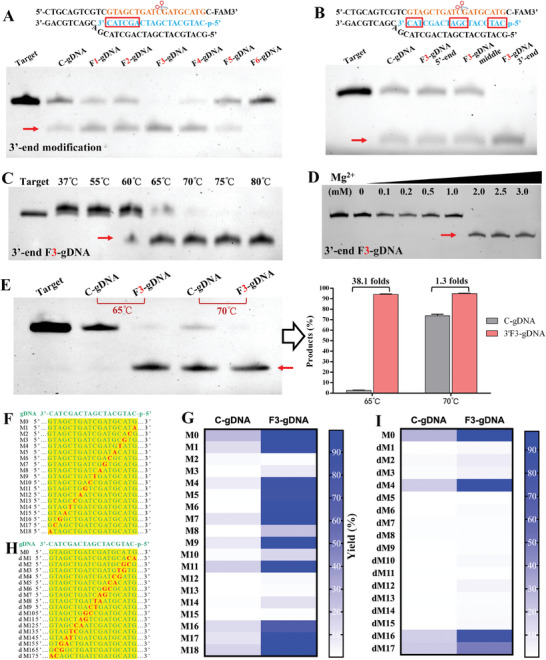
The outstanding performance of FATE system. A) Optimization for the number of 2′F‐nucleotides. B) Comparison of 2′F‐modification regions on gDNA. C) Reaction temperature optimization for the FATE. D) Magnesium concentration optimization for the FATE. E) Comparison of dsDNA cleavage activity between FATE and canonical gDNA/TtAgo system. F) The targets with single‐base mismatch. G) Specificity of the FATE for single‐base mismatched targets. H) The targets with double‐mismatched bases. I) Specificity of the FATE for double‐mismatched targets. The red arrows refer to the cleavage product. The heat maps of Figure 3G,I were plotted according to the grayscale ratio of product to substrate in Figure S7 (Supporting Information).

For best results, we optimized the 2′F‐modification strategy. Data showed that for 3′‐end modification, the TtAgo activity increases with the number of 2′F‐nucleotides and reaches the maximum when the gDNA has three 2′F‐nucleotides (**Figure** **3**A). Further, the gDNA with 3′‐end modification provided higher dsDNA cleavage activity than that with intermediate and 5′‐end modification (Figure 3B). Thus, the gDNA with three 2′F‐nucleotides at 3′‐end (**F3‐gDNA** for short) was regarded as the optimal modification strategy, namely the F3‐gDNA assisted TtAgo activity enhancement (**FATE)**.

Furthermore, we optimized reaction temperature (directly related to activity) and magnesium concentration (necessary for TtAgo activation) for FATE reaction. Data showed that the FATE reaction reaches the maximum dsDNA cleavage activity under 2 µM magnesium at 70 °C (Figure 3C,D; Figure S5, Supporting Information). The FATE reaction can provide approximately 40‐fold activity enhancement comparing to the canonical gDNA/TtAgo when it was performed at 65 °C (Figure 3E). Surprisingly, besides activity enhancement, the FATE decreased the minimum reaction temperature from 65 to 60°C (Figure 3C; Figure S6, Supporting Information). In addition, the specificity of the FATE system was analyzed by using targets with mismatches (Table S3, Supporting Information). Data showed that the FATE system can discriminate single‐base mismatch (Figure 3F,G; Figures S6 and S7, Supporting Information), under circumstances that the target DNA had mismatch at the 2nd, 3rd, 8th, 10th, 12th, 13th, 14th, or 15th nucleotide. When the target DNAs had two consecutively mismatched nucleotides, the FATE system showed little activity to them, except for the targets with 4–5th, 16–17th, or 17–18th mismatches (Figure 3H,I; Figures S6 and S7, Supporting Information). Except for these discriminable mismatch sites, the FATE system still showed significantly higher activity than the canonical gDNA/TtAgo system (Figure S8, Supporting Information). These data collectively demonstrated that the FATE system is super‐active and highly specific for dsDNA cleavage.

### 2.4 Principle for FATE Assisted Nucleic Acid Testing

**Figure 4:**
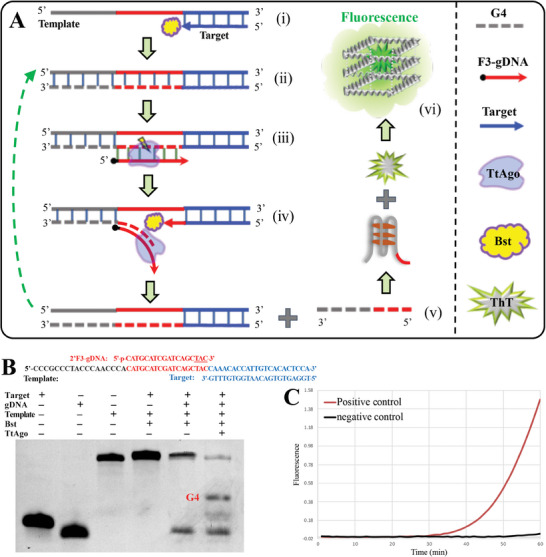
Principle of the FAST assay. A) Principle of the FAST. B) FAST triggered G4 formation. The G4 can be produced only if TtAgo and Bst DNA polymerase are present at the same time indicating that they work cooperatively and successfully. C) Real‐time FAST assay based on ThT. Due to the existence of a complementary G4 motif in template, the FATE combined with Bst DNA polymerase can extend and nick the target repeatedly, resulting in continuous generation of G4. Subsequently, the G4 lights up the ThT, and thus realizes real‐time fluorescence.

By taking advantage of the high‐efficient FATE system, we developed a novel isothermal amplification method, namely FATE assisted nucleic acid testing (**FAST**). As shown in **Figure** **4**A, the FAST assay is carried out by using target‐specific template, universal F3‐gDNA, TtAgo, and Bst DNA polymerase at 65–70 °C. The target‐specific template consists of a target‐specific 3′‐end (complementary to the target) and a universal 5′‐end, which are composed of a complementary G‐quadruplex (G4) motif and a gDNA motif. The FAST assay is performed at 65–70 °C. At this temperature, both the TtAgo and Bst DNA polymerase can provide relatively high activity.

The FAST assay realizes nucleic acid testing via following steps. i) The single‐strand targets (such as microRNA) hybridize with the FAST templates. ii) The Bst DNA polymerases trigger extension, generating specific dsDNA containing F3‐gDNA binding site. iii) This specific dsDNA is subsequently nicked by the FATE. iv) The Bst DNA polymerases bind to the nick sites, subsequently trigger the 3‐end extension and strand displacement, as a result generating large amounts of single strand G4 v). Since G4 can bind thioflavin T (ThT) and light it up (fluorescence enhancement),^[^ ^24^ ^]^ the ThT is added to the FAST reaction mixture and used to indicate the reaction process vi). Data showed that the FAST assay works expectedly, generating large amounts of G4 (Figure 4B) and realizing real‐time analysis after ThT addition (Figure 4C). Notably, the only difference among FAST assays is the template. Thus, a new FAST assay can be easily established, thereby facilitating the experiment design and clinical application.

### 2.5 Performance of FAST in Microrna Detection

**Figure 5:**
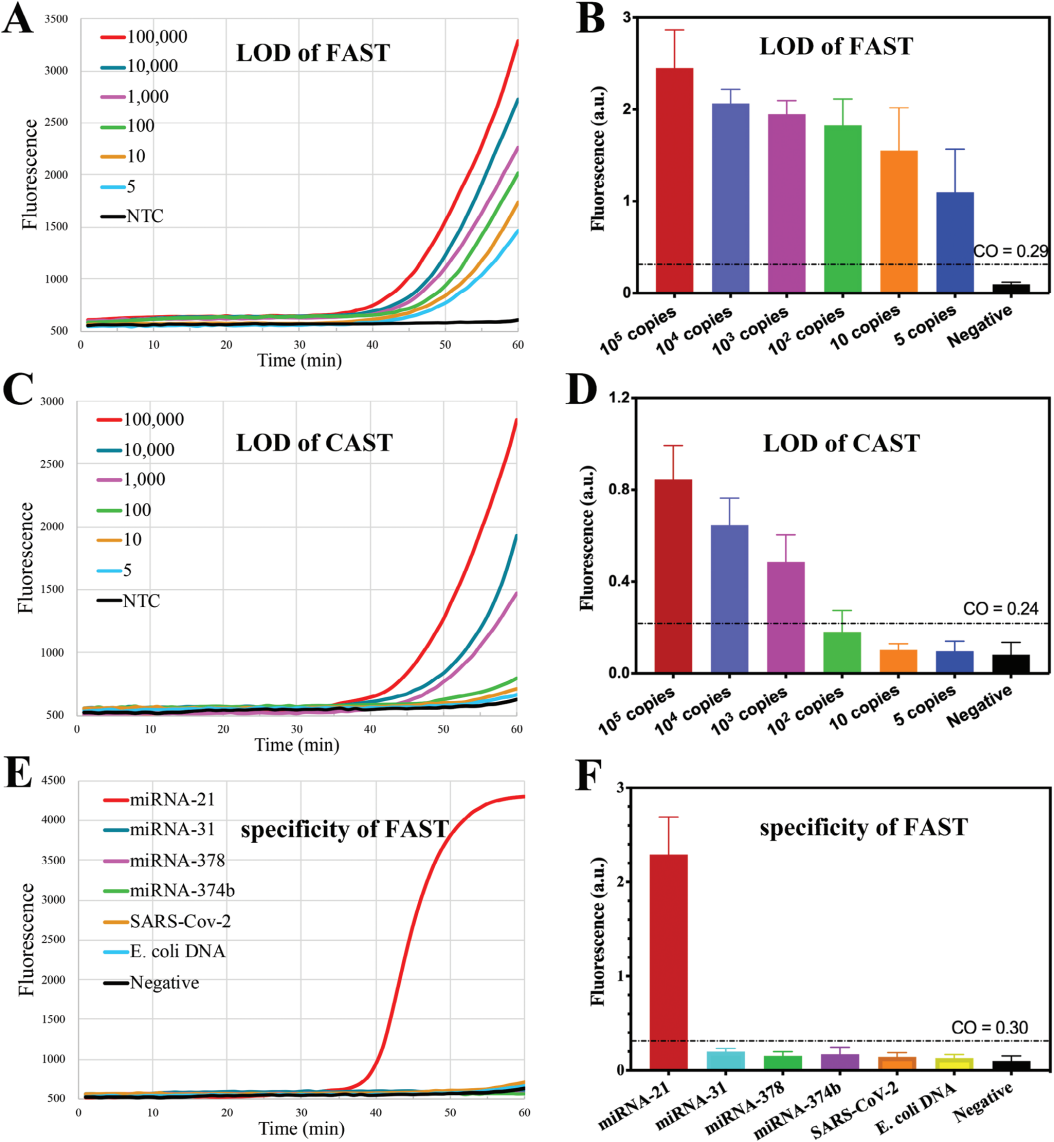
FAST for microRNA detection. A) LOD of FAST. The FAST assays were carried out by using 1 to 10^5^ copies of chemically synthesized miR‐21and real‐timely monitored by isothermal fluorometer. B) The LOD of FAST based on endpoint fluorescence. The endpoint fluorescence was measured by fluoroanalyzer. (C and D) LOD of CAST. The CAST refers to canonical gDNA/TtAgo assisted isothermal nucleic acid testing. (E and F) Specificity of FAST assay. Three independent replicates were performed. The endpoint fluorescence was used for positive recognition and the cutoff value (CO) was defined as three times of the endpoint fluorescence value of negative control.

In order to investigate the FAST performance, we adopted microRNA‐21 (miR‐21) as the target for illustration, which is a high‐profile cancer‐promoting microRNA, involved in tumor proliferation, apoptosis, and invasion.^[^ ^25^ ^]^ According to the FAST principle, a miR‐21‐specific template was first constructed (Table S4, Supporting Information). Then, the limit of detection (LOD) of FAST assay was carried out by using chemically synthesized miR‐21. The endpoint fluorescence was used for positive recognition. Data showed that our FAST assay can detect the miR‐21 up to 5 copies in 20 µL reaction mixture (**Figure** **5**A,B; Figure S9, Supporting Information), while the canonical gDNA/TtAgo assisted isothermal nucleic acid testing (CAST) showed poor LOD (up to 100 copies, Figure 5C,D). Further, the specificity of FAST was investigated by using a set of nonspecific nucleic acids, including miR‐31, miR‐378, miR‐374b, SARS‐Cov‐2 RNA, and *E. coli* DNA. It is evident that the FAST assay can resist nonspecific nucleic acid disruption during 60 min incubation (Figure 5E,F). These data demonstrated that the FAST is ultra‐sensitive and high‐specific for microRNA detection.

### 2.6 FAST for Real Sample Detection

**Figure 6:**
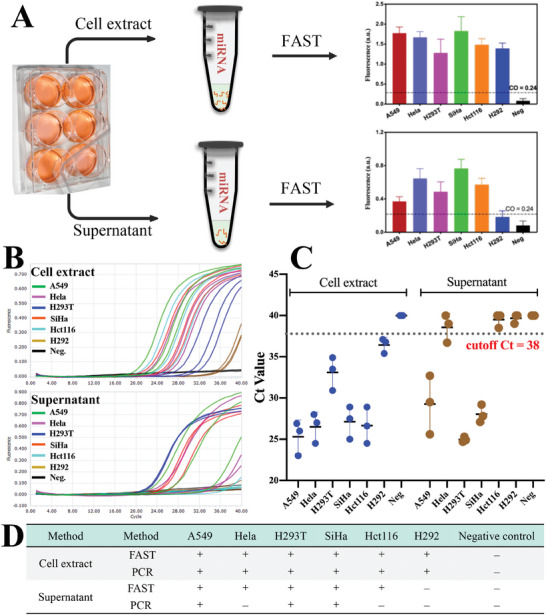
Practical application of FAST assay. A) FAST assay for miR‐21 detection in cell extract and culture supernatant. B) RT‐PCR for miR‐21 detection. C) Ct distribution of the cell extract and culture supernatant from Figure 6B. D) Performance comparison between FAST and PCR. All the PCR‐positive samples were correctly identified by FAST assay, and two PCR‐negative samples were successfully identified as positive by FAST.

To validate the practicability of FAST, several kinds of miR‐21producing cell line (A549, Hela, H293T, SiHa, Hct116, and H292) and their culture supernatant were analyzed by both FAST assay and stem–loop RT‐PCR (a canonical microRNA detection method).^[^ ^26^ ^]^ Data showed that (**Figure** **6**) all the cell extracts were identified as miR‐21 positive by both FAST and RT‐PCR, offering completely identical results. Further, for culture supernatant sample, the FAST assay offered a higher positive rate (five out of six) than the RT–PCR (three out of six). Besides microRNA detection, the FAST can also be applied to long‐chain nucleic acid detection (dsDNA, for example) by combining it with proper fragmenting pretreatment of nucleic acid (Figure S10, Supporting Information). These results collectively indicated that the FAST assay has high sensitivity and fine practicability for nucleic acid detection.

## 3 Discussion

It is well‐known that the DNA‐guided TtAgo system has poor activity for dsDNA cleavage under relatively low temperature, consequentially limiting its potential for practical application, such as nucleic acid detection and gene editing. Based on the well‐defined cleavage mechanism of TtAgo‐guide‐target ternary complex,^[^ ^13^, ^27^ ^]^ we reasonably inferred that the poor competitive advantage of canonical gDNA may be the underlying cause for the poor dsDNA cleavage activity. Since the canonical gDNA has identical Tm (namely nucleotide matching capability) to the homologous strand of target dsDNA, it is hard to invade the dsDNA, and further interrupts formation of TtAgo‐guide‐target ternary complex. Based on this mechanism understanding, we hypothesized that enhancing the binding affinity of gDNA by chemical modification may be an effective way to increase the dsDNA cleavage activity of TtAgo.

According to our hypothesis, we adopted 2′F‐, 2′OMe‐, PS‐, LNA‐, and MGB‐modification to enhance the gDNA's Tm and TtAgo's dsDNA cleavage activity. We found that the 2′F‐, 2′OMe‐, PS‐ and LNA‐modified gDNA showed higher Tm than their natural counterpart as expected, and consequently enhanced the dsDNA cleavage activity of TtAgo significantly, thus demonstrating our hypothesis. Notably, although the 2′F‐modified gDNA has merely moderate Tm increase, it contributed the greatest gains in enhancing dsDNA cleavage activity, indicating that factors other than Tm implicate in the activity enhancement.

Previous studies reported that the 2′F‐modification is the best mimic of 2′OH in terms of size and charge, it can increase the Tm and help the guide bind to the RISC complex.^[^ ^28^ ^]^ Further, the 2′F modification favors the 3′‐endo conformation, which leads to an A‐form helix (the preferably topological structure for TtAgo‐guide‐target ternary complex).^[^ ^15^ ^]^ Thus, the 2′F‐gDNA was given high expectations. As expected, the 2′F‐modification not only increased the Tm of gDNA notably, but also enhanced its binding affinity to TtAgo significantly. The Tm elevation can provide ability for gDNA to invade into the target dsDNA, while the binding affinity enhancement can further facilitate the formation of TtAgo‐guide‐target ternary complex. Consequently, the 2′F‐gDNA offered the most effective activity enhancement for TtAgo‐mediated dsDNA cleavage.

It's worth noting that the MGB‐modified gDNA has most significant Tm increase, but it causes activity reduction. Structural studies have revealed that A‐form DNA helix is the proper conformation for TtAgo‐mediated target cleavage.^[^ ^13^ ^]^ Whereas, the crescent‐shaped MGB prefers to bind to B‐form DNA (iso‐helical to the minor groove of B‐form DNA).^[^ ^19^ ^]^ Thus, the MGB‐modification may interrupt formation of to A‐form gDNA, thereby suppressing cleavage activity of TtAgo. This data indicated that the molecular interactions between gDNA and target DNA are also crucial for TtAgo activity. Fortunately, the 2′F‐modified gDNA not only provided Tm and binding affinity increase, but also offered appropriate molecule interaction between 2′F‐gDNA and amino acid residues, as a result enhancing TtAgo's activity significantly. Especially, the F3‐gDNA/TtAgo system (FATE) provided ≈40‐fold activity enhancement compared to the canonical counterpart and decreased its minimum reaction temperature from 65 to 60 °C. These performance improvements provided the practical application potentials to TtAgo.

To our knowledge, previous molecular diagnosis applications of TtAgo were focused on target recognition and signal output,^[^ ^20^, ^29^, ^30^, ^31^, ^32^, ^33^ ^]^ and TtAgo‐mediated nucleic acid amplification has not been reported. To take advantage of the FATE, we developed a novel isothermal amplification technology, named FAST (Figure 4), which is realized by using the 2′F‐gDNA for target recognition, TtAgo for target nicking, and Bst DNA polymerase for extension and strand‐displacement. Since the ThT can bind to G‐quadruplex (G4) and lead to dramatic fluorescence enhancement.^[^ ^24^, ^34^ ^]^ The ThT was adopted to indicate the G4 products generated by FAST assay. Thanks to the outstanding dsDNA cleavage activity of FATE, our FAST assay achieves attomolar sensitivity for microRNA detection. Due to its ingenious scheme (only requiring two oligonucleotides: a template DNA and a guide DNA), the FAST theoretically has high specificity. Because the more kinds of oligonucleotides there are, the larger possibility for primer‐dimer and nonspecific amplification. As expected, our FAST provides ultralow background amplification and realizes satisfied specificity. Furthermore, the FAST can realize different targets detection by using a universal 2′F‐gDNA, implying its convenience, economy, and robustness in practical application. In addition, when a long‐chain nucleic acid was fragmented properly, it also can be analyzed by FAST (Figure S10, Supporting Information). These data demonstrated the high sensitivity and fine practicability of FAST assay.

## 4 Conclusion

In conclusion, we found that the underlying cause for the poor dsDNA cleavage activity of TtAgo is the weak intermolecular force among gDNA, TtAgo, and target DNA, which impedes TtAgo‐guide‐target ternary complex formation. Based on this mechanism understanding, we have developed the atom‐modification‐based FATE strategy (the 2′‐hydrogen of gDNA is substituted with fluorine atom) to increase the Tm and binding affinity of gDNA, thereby realizing unprecedented activity enhancement of TtAgo. To take advantage of the FATE, we established a novel, ultrasensitive, and high‐specific isothermal amplification technology (FAST), thereby providing a useful tool for rapid molecular diagnosis. Further, the outstanding FATE system brings a gene editing potential to other argonautes working at moderate temperature, such as Clostridium butyricum argonaute.

## 5 Experimental Section

### Materials

The reagents including DNA oligonucleotides, dNTPs, acrylamide/methylene acrylamide (29:1), 10% SDS‐PAGE color gel rapid preparation kit, potassium chloride, ammonium sulfate, magnesium sulfate, 20% Tween, and 10× TBE buffer were purchased from Shanghai Sangon Biotech (Shanghai, China). The duplex‐specific nuclease (DSN) and microRNA Extraction kit were purchased from Bolaz Biotechnology (Nanjing, China). The Bst DNA polymerase 2.0 and 10× Isothermal Amplification Buffer were purchased from New England Biolabs (Beijing, China).

### TtAgo Expression and Purification

The expression plasmid of TtAgo (Thermus thermophilus argonaute, pWUR702#53 079) was purchased from HedgehogBio Technology Co., Ltd. (Shanghai, China), and its sequence was shown in Table S1 (Supporting Information). The expression and purification steps were referred to the previous publication^[^ ^20^ ^]^ and briefly described as follows. The plasmid was transformed into the BL21 competent cells, which were then spread on solid LB plates for 12–16 h of incubation at 37 °C. The colonies were further cultured in liquid LB (containing 50 µg/ml ampicillin) at 37 °C until the culture's OD600 increased to 0.7. Then IPTG (final concentration is 1 mm) was added for induction expression at 20 °C for 16 h overnight. The induced cells were harvested by centrifugation and lysed by sonication in lysis buffer (20 mm Tris–HCl pH 7.5, 250 mm NaCl, 5 mm imidazole, supplemented with protease inhibitor cocktail). The soluble lysate was filtered 0.2 µm filter membrane, then loaded on a nickel column for protein purification. The column was washed with a buffer containing 20 mm Tris–HCl pH 7.5, 250 mm NaCl, and 30 mm imidazole. The bound proteins were eluted by increasing the imidazole concentration of wash buffer to 250 mm. Next, the eluted protein was dialyzed in a buffer with 20 mM HEPES pH 7.5, 250 mm KCl, 1 mM DTT, and 1 mg TEV protease. Finally, the purified TtAgo was identified by SDS‐PAGE (Figure S1, Supporting Information) and diluted to a final concentration of 5 µm and stored at –80 °C.

### Guide DNA Preparation

The canonical and chemically modified guide DNAs (including 2′F‐, 2′OMe‐, LNA‐, MGB‐ and phosphorothioate‐modified gDNA) were adopted to activate TtAgo‐mediated cleavage assay in this study. These guide DNAs and the corresponding targets listed in Table S2 (Supporting Information) were synthesized by Shanghai Sangon Biotech (Shanghai, China).

### gDNA/TtAgo Mediated Cleavage Assay

The 2 µM TtAgo, 10 µm canonical or modified guide DNA, and 1x reaction buffer (20 mm Tris‐HCl (pH 8.4), 25 mm KCl, 10 mM (NH_4_)_2_SO_4_, 2 mm MgCl_2_) were mixed and incubated at 75 °C for 30 min for gDNA/TtAgo assembly (tenfold dilution for application). During the cleavage assay, target ssDNA or dsDNA (0.1–0.5 µm) was incubated with the 10‐fold‐diluted gDNA/TtAgo complex in 1x reaction buffer at 60–80 °C for 30 min. The reaction was stopped with loading buffer (2X) containing saturated urea and EDTA and denatured at 95 °C for 5 min. Finally, the products were analyzed by 12% denaturing polyacrylamide gel electrophoresis (PAGE), stained with GelRed (San‐gon Biotech, Shanghai, China), and visualized by the ChemiDocTM XRS Imaging System (Bio‐Rad Laboratories Co., Ltd., California, USA).

### Melting Temperature Analysis

The melting temperature (Tm) of canonical gDNA and chemically modified gDNA was analyzed by using intercalating dye (SYBR Green I) based on the fundamental thermodynamic property of DNA. Tm analysis was carried out on LightCycler 96 System by ramping the temperature from 55 to 95 °C at 0.07 °C s^−1^. The melting curve was obtained from the continuous monitoring of fluorescence during temperature changes.

### Biomolecular Interaction Analysis

The binding affinity between TtAgo and 2′F‐modified gDNA was analyzed by using biomolecular interaction analysis and carrying out on Octet K2 system (Pall ForteBio LLC, California, USA). The analysis was followed with the manufacturer's instructions, briefly including: Step 1, incubate the streptavidin‐modified probes in 1x reaction buffer for 10 min; step 2, loading the streptavidin probe to bind biotin‐modified gDNA (chemically synthesized by Shanghai Sangon Biotech); step 3, wash the probe with 1x reaction buffer; step 4, capture the TtAgo by the gDNA‐linked probe; step 5, dissociate the TtAgo by using 1x reaction buffer. Finally, the binding affinity was calculated on the basis of association and dissociation processes.

### PAGE Analysis

The 12% denaturing PAGE gel (10 mL) was prepared with 4 mL acrylamide‐bisacrylamide solution (29:1, 30% w/v), 4.8 g urea, 1.0 mL 10 × TBE, 5 µL TEMED, and 0.10 mL 10% APS, and proper amount of ddH_2_O. The 12% non‐denaturing PAGE gel was the same as the denaturing PAGE gel except for without urea addition. Then, 5 µL of reaction products were mixed with 5 µL denaturing loading buffer (2X) or 1 µL loading buffer (6X). The products were added to gel wells for electrophoresis at 180 V for 30 min in 1 × TBE buffer at room temperature. After electrophoresis, the gel was stained with GelRed and visualized by the ChemiDoct XRS Imaging System (Bio‐Rad Laboratories Co., Ltd, California, USA).

### FAST Assay

The FAST assays were carried out in a 20 µL reaction mixture containing 1x Bst isothermal amplification buffer, 0.8 m betaine, 6 mm MgSO4, 1.2 mm dNTP mixture, 0.5 µm 2′F‐gDNA and 0.5 µm FAST template, 8 U Bst DNA polymerase, 0.1 µm TtAgo, 1 mm KCl, 30 µm ThT and various concentrations of target (miR‐21 for example). The oligos used for FAST assay were listed in Table S4 (Supporting Information). The FAST assays were typically performed at 65 °C for 60 min and real‐time monitored by Light‐Cycler 96 (Roche diagnostics, Mannheim, Germany), which was set to measure the fluorescence intensity in every assay tube at one‐minute intervals. After real‐time analysis, the products were further analyzed by PAGE.

### FAST for miR‐21 Detection

To investigate the practical application of FAST, chemically synthesized miR‐21 (Table S4, Supporting Information) and cell‐extracted microRNA were adopted as the target for illustration. The chemically synthesized miR‐21 was dissolved with DEPC‐treated H_2_O, then serially diluted to 10^6^, 10^5^, 10^4^, 10^3^, 10^2^, 10^1^ and 10^0^ copies µL^−1^, which were applied to sensitivity, specificity, and linearity evaluation. The A549 cells were cultured in DMEM medium supplying with 10% calf serum. Then, microRNA was extracted from the A549 cell and its culture supernatant respectively by using MiPure Cell/Tissue miRNA Kit (Vazyme Biotech, Nanjing, China), and used for FAST‐based miR‐21 detection. In addition, we designed canonical stem‐loop primers for PCR‐based miR‐21 detection, which includes reverse transcription and amplification steps. The reverse transcription was performed with 4 µL 5x Hifair Buffer, 10 U Hifair III reverse transcription, 2 U RNase inhibitor, 10 mm dNTP Mix, 0.5 um stem‐loop template, 2 µL extracted RNA and ddH_2_O (total reaction mixture 20 µL) at 25 °C/5 min, 55 °C/30 min, and 85 °C/5 min. The PCR was performed with 10 µL 2x AceQ qPCR Master Mix (Yisheng Biotech, Shanghai, China), 0.5 µM F‐primer, 0.5 µM B‐primer, 2 µL template and ddH_2_O (step 1: 95 °C/5 min, step 2: 95 °C/15 s, 60 °C/30 s, and 40 cycles).

### FAST for dsDNA Detection

To realize FAST‐based dsDNA detection, DSN was employed for dsDNA fragmenting. In this study, a plasmid containing 1518 bp of type 16 Human papillomavirus L1 gene (pHPV‐DNA) was fragmented by 1 mg mL^−1^ DSN at 37 °C for 0, 5, 10, 15, 20, 25, 30, 45, and 60 min, followed by inactivation at 95 °C for 5 min. Then, the FAST assays were carried out with 2 µL of the fragmented products.

## Conflict of Interest

The authors declare no conflict of interest.

## Supporting information

Supporting Information

## Data Availability

The data that support the findings of this study are available in the supplementary material of this article.

## References

[1] Z. Xu , D. Chen , T. Li , J. Yan , J. Zhu , T. He , R. Hu , Y. Li , Y. Yang , M. Liu , Nat. Commun. 2022, 13, 6480. 36309521 10.1038/s41467-022-34086-y PMC9617605

[2] R. Zheng , L. Zhang , R. Parvin , L. Su , J. Chi , K. Shi , F. Ye , X. Huang , Adv. Sci. 2023, 10, e2300195. 10.1002/advs.202300195 PMC10477906 37356052

[3] W. Zhang , Y. Mu , K. Dong , L. Zhang , B. Yan , H. Hu , Y. Liao , R. Zhao , W. Shu , Z. Ye , Y. Lu , C. Wan , Q. Sun , L. Li , H. Wang , X. Xiao , Nucleic Acids Res. 2022, 50, 12674. 36484104 10.1093/nar/gkac1144 PMC9825152

[4] Y. Wu , W. Luo , Z. Weng , Y. Guo , H. Yu , R. Zhao , L.i Zhang , J. Zhao , D. Bai , X.i Zhou , L. Song , K. Chen , J. Li , Y. Yang , G. Xie , Nucleic Acids Res. 2022, 50, 11727. 36318259 10.1093/nar/gkac886 PMC9723625

[5] Y. Qin , Y. Li , Y. Hu , Trends Biotechnol. 2022, 40, 910. 35418313 10.1016/j.tibtech.2022.03.006

[6] A. Kuzmenko , A. Oguienko , D. Esyunina , D. Yudin , M. Petrova , A. Kudinova , O. Maslova , M. Ninova , S. Ryazansky , D. Leach , A. A. Aravin , A. Kulbachinskiy , Nature 2020, 587, 632. 32731256 10.1038/s41586-020-2605-1

[7] E. A. Hunt , T. C. Evans Jr. , N. A. Tanner , PLoS One 2018, 13, e0203073. 30157272 10.1371/journal.pone.0203073 PMC6114923

[8] G. Luo , H. He , D. Wang , S. Liu , S. Tian , M. Chen , Q. Wang , C. Zhao , Z. Leng , L. Hou , X. Guo , Clin. Chem. 2023, 69, 363. 36807661 10.1093/clinchem/hvac199

[9] G. Luo , T. Yi , Q. Wang , B. Guo , L.i Fang , G. Zhang , X. Guo , Biosens. Bioelectron. 2021, 184, 113239. 33857727 10.1016/j.bios.2021.113239

[10] G. Luo , J. Zhang , M. Yang , H. He , Z. Huang , J. Mater. Chem. B 2021, 9, 5636. 34196647 10.1039/d1tb00428j

[11] J. W. Hegge , D. C. Swarts , S. D. Chandradoss , T. J. Cui , J. Kneppers , M. Jinek , C. Joo , J. van der Oost , Nucleic Acids Res. 2019, 47, 5809. 31069393 10.1093/nar/gkz306 PMC6582352

[12] Y. Wang , S. Juranek , H. Li , G. Sheng , G. S. Wardle , T. Tuschl , D. J. Patel , Nature 2009, 461, 754. 19812667 10.1038/nature08434 PMC2880917

[13] G. Sheng , H. Zhao , J. Wang , Y.u Rao , W. Tian , D. C. Swarts , J. van der Oost , D. J. Patel , Y. Wang , Proc. Natl. Acad. Sci. U S A 2014, 111, 652. 24374628 10.1073/pnas.1321032111 PMC3896195

[14] A. Khvorova , J. K. Watts , Nat. Biotechnol. 2017, 35, 238. 28244990 10.1038/nbt.3765 PMC5517098

[15] D. A. Glazier , J. Liao , B. L. Roberts , X. Li , K.a Yang , C. M. Stevens , W. Tang , Bioconj. Chem. 2020, 31, 1213. 10.1021/acs.bioconjchem.0c00060 32227878

[16] S. T. Crooke , B. F. Baker , R. M. Crooke , X. H. Liang , Nat. Rev. Drug Discov. 2021, 20, 427. 33762737 10.1038/s41573-021-00162-z

[17] J. K. Watts , Chem. Commun. 2013, 49, 5618. 10.1039/c3cc40340h 23682352

[18] M. Robbins , A. Judge , L. Liang , K. McClintock , E. Yaworski , I. MacLachlan , Mol. Ther. 2007, 15, 1663. 17579574 10.1038/sj.mt.6300240

[19] I. V. Kutyavin , Nucleic Acids Res. 2000, 28, 655. 10606668 10.1093/nar/28.2.655 PMC102528

[20] J. Song , J. W. Hegge , M. G. Mauk , J. Chen , J. E. Till , N. Bhagwat , L. T. Azink , J. Peng , M. Sen , J. Mays , E. L. Carpenter , J. van der Oost , H. H. Bau , Nucleic Acids Res. 2020, 48, e19. 31828328 10.1093/nar/gkz1165 PMC7038991

[21] D. C. Guenther , S. Mori , S. Matsuda , J. A. Gilbert , J. L. S. Willoughby , S. Hyde , A. Bisbe , Y. Jiang , S. Agarwal , M. Madaoui , M. M. Janas , K. Charisse , M. A. Maier , M. Egli , M. Manoharan , J. Am. Chem. Soc. 2022, 144, 14517. 35921401 10.1021/jacs.2c01679 PMC9389587

[22] E. Malek‐Adamian , J. Fakhoury , A. E. Arnold , S. Martinez‐Montero , M. S. Shoichet , M. J. Damha , Nucleic Acid Ther. 2019, 29, 187. 31084536 10.1089/nat.2019.0792 PMC6686699

[23] D. Placido , B. Brown , K. Lowenhaupt , A. Rich , A. Athanasiadis , Structure 2007, 15, 395. 17437712 10.1016/j.str.2007.03.001 PMC2082211

[24] A. Renaud de la Faverie , A. Guedin , A. Bedrat , L. A. Yatsunyk , J. L. Mergny , Nucleic Acids Res. 2014, 42, e65. 24510097 10.1093/nar/gku111 PMC4005661

[25] J. Wei , H. Wang , X. Gong , Q. Wang , H. Wang , Y. Zhou , F. Wang , Nucleic Acids Res. 2020, 48, e60. 32347938 10.1093/nar/gkaa250 PMC7261173

[26] C. Chen , Nucleic Acids Res. 2005, 33, e179. 16314309 10.1093/nar/gni178 PMC1292995

[27] G. Sheng , T. Gogakos , J. Wang , H. Zhao , A. Serganov , S. Juranek , T. Tuschl , D. J. Patel , Y. Wang , Nucleic Acids Res. 2017, 45, 9149. 28911094 10.1093/nar/gkx547 PMC5587774

[28] M. Manoharan , A. Akinc , R. K. Pandey , J. Qin , P. Hadwiger , M. John , K. Mills , K. Charisse , M. A. Maier , L. Nechev , E. M. Greene , P. S. Pallan , E. Rozners , K. G. Rajeev , M. Egli , Angew. Chem. Int. Ed. Engl. 2011, 50, 2284. 21351337 10.1002/anie.201006519 PMC3516925

[29] S. Shin , Y. Jung , H. Uhm , M. Song , S. Son , J. Goo , C. Jeong , J.‐J. Song , V. N. Kim , S. Hohng , Nat. Commun. 2020, 11, 6033. 33247115 10.1038/s41467-020-19865-9 PMC7699633

[30] Q. Lin , G. Han , X. Fang , H. Chen , W. Weng , J. Kong , Anal. Chem. 2022, 94, 11290. 35894425 10.1021/acs.analchem.2c01945

[31] Z. Wang , Z. Wang , F. Zhang , L. Wu , Front. bioeng. biotechnol. 2023, 11, 1221943. 37583711 10.3389/fbioe.2023.1221943 PMC10424790

[32] J. Fang , C. Yuan , X. Luo , Z. He , W. Fu , Talanta 2024, 266, 125034. 37597338 10.1016/j.talanta.2023.125034

[33] Q. Lin , X. Ye , H. Chen , X. Fang , H. Chen , J. Kong , Anal. Chem. 2024, 96, 620. 38170960 10.1021/acs.analchem.3c05263

[34] S. Jing , Q. Liu , Y. Jin , L.i B. D. G‐Quadruplex , Anal. Chem. 2021, 93, 1333. 33347269 10.1021/acs.analchem.0c02637

