# Supplementary material for: Atom‐Modified gDNA Enhances Cleavage Activity of TtAgo Enabling Ultra‐Sensitive Nucleic Acid Testing
Source: Adv Sci (Weinh). 2024 May 10;11(28):2403120. doi: 10.1002/advs.202403120 (PMC11267260; doi:10.1002/advs.202403120)
Supplement: Supplementary file 1 — Supporting Information [file ADVS-11-2403120-s001.docx]

**Supplementary Information**

**Atom-modified gDNA enhances cleavage activity of TtAgo enabling** **ultra-sensitive nucleic acid testing**

**Running head:** 2’F-gDNA mediated TtAgo activity enhancement

Jun Zhang^1, 4#^, Miaomiao Chen^1#^, Huan Jiang^1^ Huifang Sun^1^, Jianing Ren^2^, Xin Yang^1^, Shanshan Liu^1^, Dongsheng Wang^3^, Jianping Liu^2*^, Daiyuan Ma^2*^; Xiaolan Guo^1*^, Guangcheng Luo^1*^

^1^ Department of Clinical Laboratory, Affiliated Hospital of North Sichuan Medical College; School of Laboratory Medicine & Translational Medicine Research Center, North Sichuan Medical College, Nanchong 637000, China.

^2^ Department of Oncology & Department of Rheumatology and Immunology, Affiliated Hospital of North Sichuan Medical College, Nanchong 637000, China.

^3^ Department of Clinical Laboratory, Sichuan Cancer Hospital, School of Medicine, University of Electronic Science and Technology of China, Chengdu 610041, China.

^4^ Key Laboratory of Bio-Resource and Eco-environment of Ministry of Education, College of Life Sciences, Sichuan University, Chengdu, Sichuan, 610064, China.

* To whom correspondence should be addressed at email: luocheng120811@126.com (Guangcheng Luo), alan5200@hotmail.com (Xiaolan Guo), mdylx@163.com (Daiyuan Ma) or ljpbr@sina.com (Jianping Liu)

^#^ These authors contributed equally to this work.

Table S1. The sequence of TtAgo expression plasmid.

| TtAgo plasmid sequence (7740 bp, 5' to 3'): |
| --- |
| *ctgaattgactctcttccgggcgctatcatgccataccgcgaaaggttttgcgccattcgatggtgtccgggatctcgacgctctcccttatgcgactcctgcattaggaagcagcccagtagtaggttgaggccgttgagcaccgccgccgcaaggaatggtgcatgcaaggagatggcgcccaacagtcccccggccacggggcctgccaccatacccacgccgaaacaagcgctcatgagcccgaagtggcgagcccgatcttccccatcggtgatgtcggcgatataggcgccagcaaccgcacctgtggcgccggtgatgccggccacgatgcgtccggcgtagaggatcgagatctcgatcccgcgaaattaatacgactcactataggggaattgtgagcggataacaattcccctctagaaataattttgtttaactttaagaaggagatataccatgggcagcagccatcatcatcatcatcacagcagcggcatgaaccaccttggaaaaacggaagtgttcctaaaccgcttcgccctacggcctctgaaccccgaggaactcaggccttggcgcctcgaggtggttctggaccctcctccggggcgggaggaggtgtatccccttctcgcccaggtggcccgccgggcggggggcgtcacggtgcgcatgggagacggcctcgcctcctggtcaccccctgaggtcctggtcttggagggcaccttggcgcggatggggcagaactacgcctaccgcctctaccccaaggggaggaggcctctggaccccaaggacccgggggagcggagcgtgctttcggccctagcccgaaggctccttcaggagcgcctcaggcgcctcgagggggtctgggtggaggggcttgcggtgtaccggagggagcacgcccgggggcccgggtggcgggtgcttgggggggcggtcttggacctttgggtctccgactcgggggcgttcctcctggaggtggaccccgcttaccgcatcctctgcgagatgtccctcgaggcctggcttgcccaaggccaccctctgcccaaacgggtccggaacgcctacgaccggcgcacctgggagcttctccggctgggagaggaagaccccaaggagctccctctcccgggcggcttgagcctcctggactaccacgctttcaagggccgcctccagggccgggaaggggggcgggtggcctgggtggcggacccaaaggatccgcgaaagccaatcccccatctgacgggccttttggtgcccgttctgaccctggaagaccttcatgaagaggagggaagcctggccctctccttgccttgggaagagcggcgtcggcggacccgggagatcgccagctggatcggccggcgcctggggctgggcacacctgaggcggtgcgcgcccaggcttaccgcttgagcatccccaagctcatgggcagaagagccgtgagcaagcctgcggacgccctccgcgtggggttttaccgggcccaggaaaccgccctggccctcttgcgcctggacggggcccaaggatggccggagtttctccggcgggccttgctccgggcctttggggcgagcggggcttccctccgtttgcacaccctccacgcccatccctcccaaggcctggctttccgcgaggcgctgcggaaggccaaggaggagggggtccaggccgtgctggtcctcaccccgcccatggcctgggaagaccgcaaccgcctgaaggcccttcttctcagggaaggccttcccagccaaatcctcaacgtccccctccgggaggaggaacgccaccgctgggagaacgccctcctgggcctcctggccaaagcggggcttcaggtggtggccctgagcggcgcctatccggcggagctcgccgtgggctttgacgccggcggaagggagtcctttcgcttcgggggcgcggcctgcgccgtgggcggggacggcggccacctcctctggaccctccccgaggcccaggccggggagcggatcccccaggaggtggtctgggacctcttggaggagaccctctgggccttcagacgcaaggcggggaggcttccttcccgggtcctcctccttcgggacggccgcgtgccccaggacgagttcgccctggccttggaggcccttgcccgggaaggcatagcctacgacttggtttcggtgcgcaagtcgggtggggggcgggtctaccccgtgcaggggcgcctggcggacgggctttacgtccccttggaggacaagacctttcttctccttaccgtccaccgggacttccggggcacgccccgacccctgaagctggtgcacgaggcgggggacacgcccctcgaggccctggcccaccagattttccatctgacccgcctctacccggcgagcggtttcgccttcccccggcttcccgctccccttcacctggccgaccgcctggtgaaggaggtgggccggttggggatccgtcacctcaaggaggtggaccgggaaaagctcttcttcgtttagtgaggatccggctgctaacaaagcccgaaaggaagctgagttggctgctgccaccgctgagcaataactagcataaccccttggggcctctaaacgggtcttgaggggttttttgctgaaaggaggaactatatccggatatcccgcaagaggcccggcagtaccggcataaccaagcctatgcctacagcatccagggtgacggtgccgaggatgacgatgagcgcattgttagatttcatacacggtgcctgactgcgttagcaatttaactgtgataaactaccgcattaaagcttatcgatgataagctgtcaaacatgagaattcttgaagacgaaagggcctcgtgatacgcctatttttataggttaatgtcatgataataatggtttcttagacgtcaggtggcacttttcggggaaatgtgcgcggaacccctatttgtttatttttctaaatacattcaaatatgtatccgctcatgagacaataaccctgataaatgcttcaataatattgaaaaaggaagagtatgagtattcaacatttccgtgtcgcccttattcccttttttgcggcattttgccttcctgtttttgctcacccagaaacgctggtgaaagtaaaagatgctgaagatcagttgggtgcacgagtgggttacatcgaactggatctcaacagcggtaagatccttgagagttttcgccccgaagaacgttttccaatgatgagcacttttaaagttctgctatgtggcgcggtattatcccgtgttgacgccgggcaagagcaactcggtcgccgcatacactattctcagaatgacttggttgagtactcaccagtcacagaaaagcatcttacggatggcatgacagtaagagaattatgcagtgctgccataaccatgagtgataacactgcggccaacttacttctgacaacgatcggaggaccgaaggagctaaccgcttttttgcacaacatgggggatcatgtaactcgccttgatcgttgggaaccggagctgaatgaagccataccaaacgacgagcgtgacaccacgatgcctgcagcaatggcaacaacgttgcgcaaactattaactggcgaactacttactctagcttcccggcaacaattaatagactggatggaggcggataaagttgcaggaccacttctgcgctcggcccttccggctggctggtttattgctgataaatctggagccggtgagcgtgggtctcgcggtatcattgcagcactggggccagatggtaagccctcccgtatcgtagttatctacacgacggggagtcaggcaactatggatgaacgaaatagacagatcgctgagataggtgcctcactgattaagcattggtaactgtcagaccaagtttactcatatatactttagattgatttaaaacttcatttttaatttaaaaggatctaggtgaagatcctttttgataatctcatgaccaaaatcccttaacgtgagttttcgttccactgagcgtcagaccccgtagaaaagatcaaaggatcttcttgagatcctttttttctgcgcgtaatctgctgcttgcaaacaaaaaaaccaccgctaccagcggtggtttgtttgccggatcaagagctaccaactctttttccgaaggtaactggcttcagcagagcgcagataccaaatactgtccttctagtgtagccgtagttaggccaccacttcaagaactctgtagcaccgcctacatacctcgctctgctaatcctgttaccagtggctgctgccagtggcgataagtcgtgtcttaccgggttggactcaagacgatagttaccggataaggcgcagcggtcgggctgaacggggggttcgtgcacacagcccagcttggagcgaacgacctacaccgaactgagatacctacagcgtgagctatgagaaagcgccacgcttcccgaagggagaaaggcggacaggtatccggtaagcggcagggtcggaacaggagagcgcacgagggagcttccagggggaaacgcctggtatctttatagtcctgtcgggtttcgccacctctgacttgagcgtcgatttttgtgatgctcgtcaggggggcggagcctatggaaaaacgccagcaacgcggcctttttacggttcctggccttttgctggccttttgctcacatgttctttcctgcgttatcccctgattctgtggataaccgtattaccgcctttgagtgagctgataccgctcgccgcagccgaacgaccgagcgcagcgagtcagtgagcgaggaagcggaagagcgcctgatgcggtattttctccttacgcatctgtgcggtatttcacaccgcatatatggtgcactctcagtacaatctgctctgatgccgcatagttaagccagtatacactccgctatcgctacgtgactgggtcatggctgcgccccgacacccgccaacacccgctgacgcgccctgacgggcttgtctgctcccggcatccgcttacagacaagctgtgaccgtctccgggagctgcatgtgtcagaggttttcaccgtcatcaccgaaacgcgcgaggcagctgcggtaaagctcatcagcgtggtcgtgaagcgattcacagatgtctgcctgttcatccgcgtccagctcgttgagtttctccagaagcgttaatgtctggcttctgataaagcgggccatgttaagggcggttttttcctgtttggtcactgatgcctccgtgtaagggggatttctgttcatgggggtaatgataccgatgaaacgagagaggatgctcacgatacgggttactgatgatgaacatgcccggttactggaacgttgtgagggtaaacaactggcggtatggatgcggcgggaccagagaaaaatcactcagggtcaatgccagcgcttcgttaatacagatgtaggtgttccacagggtagccagcagcatcctgcgatgcagatccggaacataatggtgcagggcgctgacttccgcgtttccagactttacgaaacacggaaaccgaagaccattcatgttgttgctcaggtcgcagacgttttgcagcagcagtcgcttcacgttcgctcgcgtatcggtgattcattctgctaaccagtaaggcaaccccgccagcctagccgggtcctcaacgacaggagcacgatcatgcgcacccgtggccaggacccaacgctgcccgagatgcgccgcgtgcggctgctggagatggcggacgcgatggatatgttctgccaagggttggtttgcgcattcacagttctccgcaagaattgattggctccaattcttggagtggtgaatccgttagcgaggtgccgccggcttccattcaggtcgaggtggcccggctccatgcaccgcgacgcaacgcggggaggcagacaaggtatagggcggcgcctacaatccatgccaacccgttccatgtgctcgccgaggcggcataaatcgccgtgacgatcagcggtccagtgatcgaagttaggctggtaagagccgcgagcgatccttgaagctgtccctgatggtcgtcatctacctgcctggacagcatggcctgcaacgcgggcatcccgatgccgccggaagcgagaagaatcataatggggaaggccatccagcctcgcgtcgcgaacgccagcaagacgtagcccagcgcgtcggccgccatgccggcgataatggcctgcttctcgccgaaacgtttggtggcgggaccagtgacgaaggcttgagcgagggcgtgcaagattccgaataccgcaagcgacaggccgatcatcgtcgcgctccagcgaaagcggtcctcgccgaaaatgacccagagcgctgccggcacctgtcctacgagttgcatgataaagaagacagtcataagtgcggcgacgatagtcatgccccgcgcccaccggaaggagctgactgggttgaaggctctcaagggcatcggtcgagatcccggtgcctaatgagtgagctaacttacattaattgcgttgcgctcactgcccgctttccagtcgggaaacctgtcgtgccagctgcattaatgaatcggccaacgcgcggggagaggcggtttgcgtattgggcgccagggtggtttttcttttcaccagtgagacgggcaacagctgattgcccttcaccgcctggccctgagagagttgcagcaagcggtccacgctggtttgccccagcaggcgaaaatcctgtttgatggtggttaacggcgggatataacatgagctgtcttcggtatcgtcgtatcccactaccgagatatccgcaccaacgcgcagcccggactcggtaatggcgcgcattgcgcccagcgccatctgatcgttggcaaccagcatcgcagtgggaacgatgccctcattcagcatttgcatggtttgttgaaaaccggacatggcactccagtcgccttcccgttccgctatcggctgaatttgattgcgagtgagatatttatgccagccagccagacgcagacgcgccgagacagaacttaatgggcccgctaacagcgcgatttgctggtgacccaatgcgaccagatgctccacgcccagtcgcgtaccgtcttcatgggagaaaataatactgttgatgggtgtctggtcagagacatcaagaaataacgccggaacattagtgcaggcagcttccacagcaatggcatcctggtcatccagcggatagttaatgatcagcccactgacgcgttgcgcgagaagattgtgcaccgccgctttacaggcttcgacgccgcttcgttctaccatcgacaccaccacgctggcacccagttgatcggcgcgagatttaatcgccgcgacaatttgcgacggcgcgtgcagggccagactggaggtggcaacgccaatcagcaacgactgtttgcccgccagttgttgtgccacgcggttgggaatgtaattcagctccgccatcgccgcttccactttttcccgcgttttcgcagaaacgtggctggcctggttcaccacgcgggaaacggtctgataagagacaccggcatactctgcgacatcgtataacgttactggtttcacattcaccacc* |

Table S2. The oligonucleotides for TtAgo mediated cleavage assay.

| Name | Sequence (5' to 3') |
| --- | --- |
| (SS guide DNA) gDNA  (1F) 2'F-gDNA  2'OMe-gDNA  LNA-gDNA  MGB-gDNA  PS-gDNA  Template DNA  5'FAM-Template DNA  Complementary DNA(Ct)  2F-gDNA  3F-gDNA  4F-gDNA  5F-gDNA  6F-gDNA  5'end of 3F-gDNA  middle of 3F-gDNA  3'end of 3F-gDNA (3F-gDNA)  5'biotin-gDNA  5'biotin-3F-gDNA  5'biotin Template DNA | 5'p-CATGCATCGATCAGCTAC  5'p-CATGCATCGATCAGCTA**/i2FC/**  5'p-CATGCATCGATCAGCTA**/i2OMeC/**  5'p-CATGCATCGATCAGCTA**iXNAC/**  5'p-CATGCATCGATCAGCTAC(**3'MGB**)  5'p-C*A*T*G*C*A*T*C*G*A*T*C*A*G*C*T*A*C  5'-CTGCAGTCGTCGTAGCTGATCGATGCATGC  5'-FAM-CTGCAGTCGTCGTAGCTGATCGATGCATGC  5'-GCATGCATCGATCAGCTACGACGACTGCAG  5'p-CATGCATCGATCAGCT**/i2FA//i2FC/**  5'p-CATGCATCGATCAGC**/i2FU//i2FA//i2FC/**  5'p-CATGCATCGATCAG**/i2FC//i2FU//i2FA//i2FC/**  5'p-CATGCATCGATCA**/i2FG//i2FC//i2FU//i2FA//i2FC/**  5'p-CATGCATCGATC**/i2FA//i2FG//i2FC//i2FU//i2FA//i2FC/**  5'p-C**/i2FA//i2FU//i2FG/**CATCGATCAGCTAC  5'p-CATGCATC**/i2FG//i2FA//i2FU/**CAGCTAC  5'p-CATGCATCGATCAGC**/i2FU//i2FA//i2FC/**  5'biotin-CATGCATCGATCAGCTAC  5'biotin-CATGCATCGATCAGC**/i2FU//i2FA//i2FC/**  5‘biotin-CTGCAGTCGTCGTAGCTGATCGATGCATGC |

Table S3. The mismatch templates used for FATE.

| Name | Sequence (5'-3') | Name | | Sequence (5'-3') | |
| --- | --- | --- | --- | --- | --- |
| gDNA  M0  M1  M2  M3  M4  M5  M6  M7  M8  M9  M10  M11  M12  M13  M14  M15  M16  M17  M18 | 5'p-CATGCATCGATCAGCTAC  CTGCAGTCGTCGTAGCTGATCGATGCATGC  CTGCAGTCGTCGTAGCTGATCGATGCATAC  CTGCAGTCGTCGTAGCTGATCGATGCACGC  CTGCAGTCGTCGTAGCTGATCGATGCGTGC  CTGCAGTCGTCGTAGCTGATCGATGTATGC  CTGCAGTCGTCGTAGCTGATCGATACATGC  CTGCAGTCGTCGTAGCTGATCGACGCATGC  CTGCAGTCGTCGTAGCTGATCGGTGCATGC  CTGCAGTCGTCGTAGCTGATCAATGCATGC  CTGCAGTCGTCGTAGCTGATTGATGCATGC  CTGCAGTCGTCGTAGCTGACCGATGCATGC  CTGCAGTCGTCGTAGCTGGTCGATGCATGC  CTGCAGTCGTCGTAGCTAATCGATGCATGC  CTGCAGTCGTCGTAGCCGATCGATGCATGC  CTGCAGTCGTCGTAGTTGATCGATGCATGC  CTGCAGTCGTCGTAACTGATCGATGCATGC  CTGCAGTCGTCGTGGCTGATCGATGCATGC  CTGCAGTCGTCGCAGCTGATCGATGCATGC  CTGCAGTCGTCATAGCTGATCGATGCATGC | F3-gDNA  M0  dM1  dM2  dM3  dM4  dM5  dM6  dM7  dM8  dM9  dM10  dM11  dM12  dM13  dM14  dM15  dM16  dM17 | 5'p-CATGCATCGATCAGC**/i2FU//i2FA//i2FC/**  CTGCAGTCGTCGTAGCTGATCGATGCATGC  CTGCAGTCGTCGTAGCTGATCGATGCACAC  CTGCAGTCGTCGTAGCTGATCGATGCGCGC  CTGCAGTCGTCGTAGCTGATCGATGTGTGC  CTGCAGTCGTCGTAGCTGATCGATGCATGC  CTGCAGTCGTCGTAGCTGATCGACACATGC  CTGCAGTCGTCGTAGCTGATCGGCGCATGC  CTGCAGTCGTCGTAGCTGATCAGTGCATGC  CTGCAGTCGTCGTAGCTGATTAATGCATGC  CTGCAGTCGTCGTAGCTGACTGATGCATGC  CTGCAGTCGTCGTAGCTGGCCGATGCATGC  CTGCAGTCGTCGTAGCTAGTCGATGCATGC  CTGCAGTCGTCGTAGCCAATCGATGCATGC  CTGCAGTCGTCGTAGTCGATCGATGCATGC  CTGCAGTCGTCGTAATTGATCGATGCATGC  CTGCAGTCGTCGTGACTGATCGATGCATGC  CTGCAGTCGTCGCGGCTGATCGATGCATGC  CTGCAGTCGTCACAGCTGATCGATGCATGC | |  |

Table S4. The oligonucleotides for miRNA-21and HPV detection.

| Name | Sequence (5'-3') |
| --- | --- |
| miRNA-21  F-primer  R-primer  stem-loop template  G-quadruplex  FAST template for miR-21  FAST template for HPV16  HPV16 positive control | UAGCUUAUCAGACUGAUGUUGA  GCCGAGTAGCTTATCAGA  CTCAACTGGTGTCGTGGA  *CTCAACTGGTGTCGTGGAGTCGGCAATTCAGTTGAG****TCAACATC***  TGGGTTGGGTAGGGCGGG  cccgccctacccaaccca-catgcatcgatcagctac-tcaacatcagtctgataagcta  cccgccctacccaaccca-catgcatcgatcagctac-acgagtctagactctgtggtat  ATACCACAGAGTCTAGACTCGT |


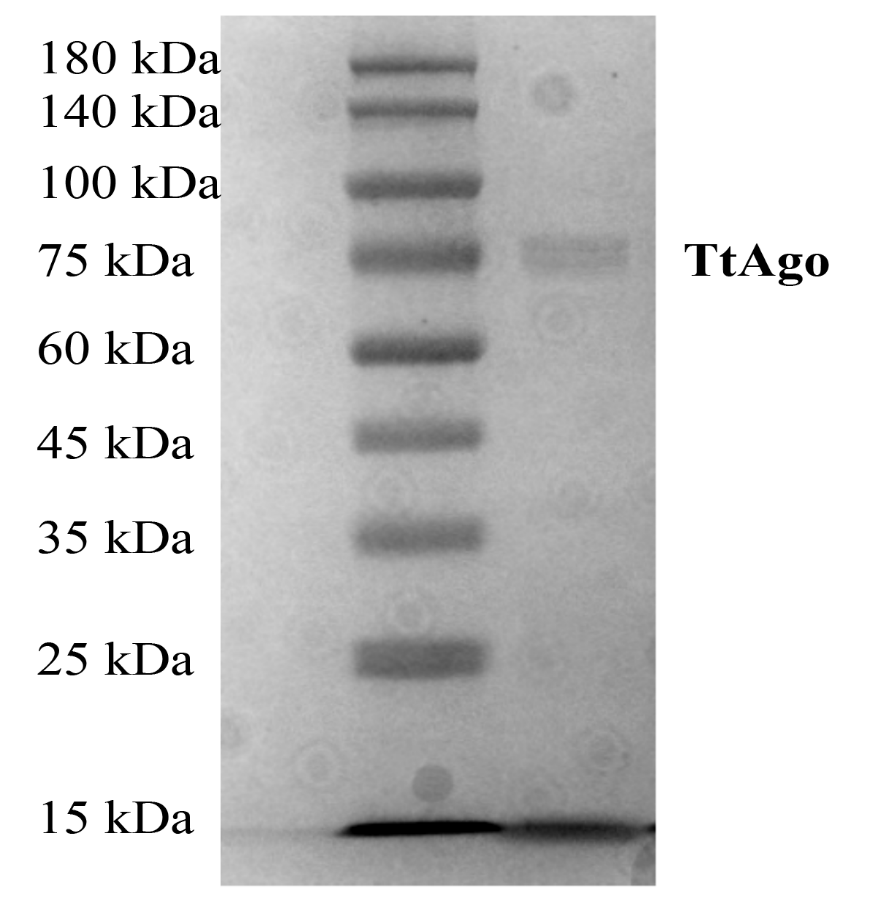


**Figure S1.** **TtAgo protein analyzed by SDS-PAGE.** The purified TtAgo protein was identified by SDS-PAGE gel electrophoresis at 180v for 45 min. The 75 kDa band was confirmed to be TtAgo protein.


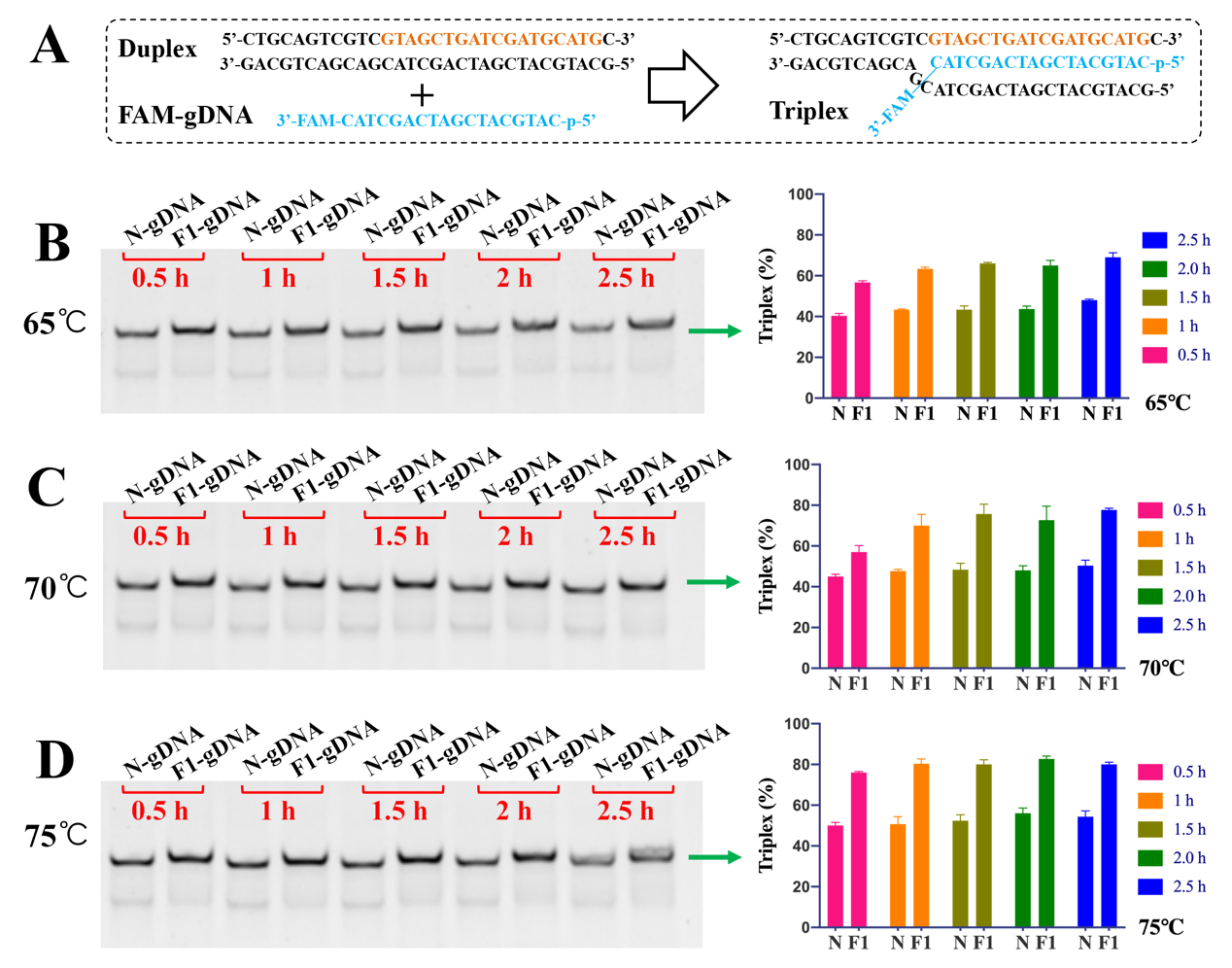


**Figure S2. Comparison strand displacement ability between F-gDNA and C-gDNA.** (A) The scheme for strand displacement analysis. (B) The strand displacement ability of gDNA at 65℃. (C) The strand displacement ability of gDNA at 70℃. (D) The strand displacement ability of gDNA at 75℃.


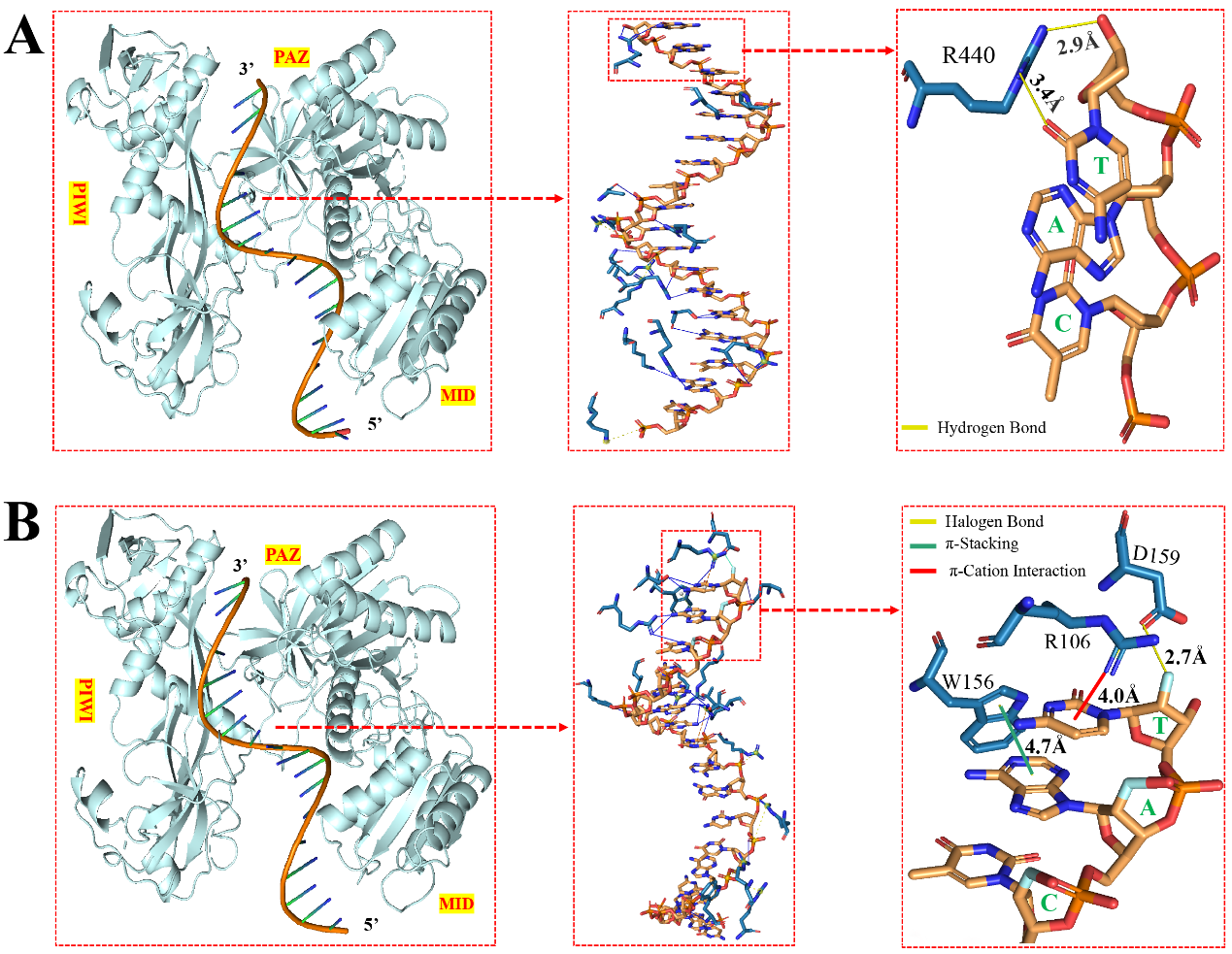


**Figure S3. Intermolecular interaction simulated by molecular docking.** (A) The interactions between the 3’-end of canonical gDNA and PAZ domain. (B) The 2’F-gDNA mediated interactions. Comparing to canonical gDNA, the 2’F-gDNA provides additional intermolecular contacts to the amino acid residues of TtAgo. For example, the 3’-end fluorine provides halogen-bond to D159, the first base at 3’-end provides π-cation interaction to R106, as well as the second base at 3’-end stacked over the W156. These additional interactions reasonably attribute to 2’F modification, and may cause structure change of the DNA-TtAgo-binary complex, thereby enhancing the stability of DNA-TtAgo-binary and improving the cleavage activity of TtAgo.


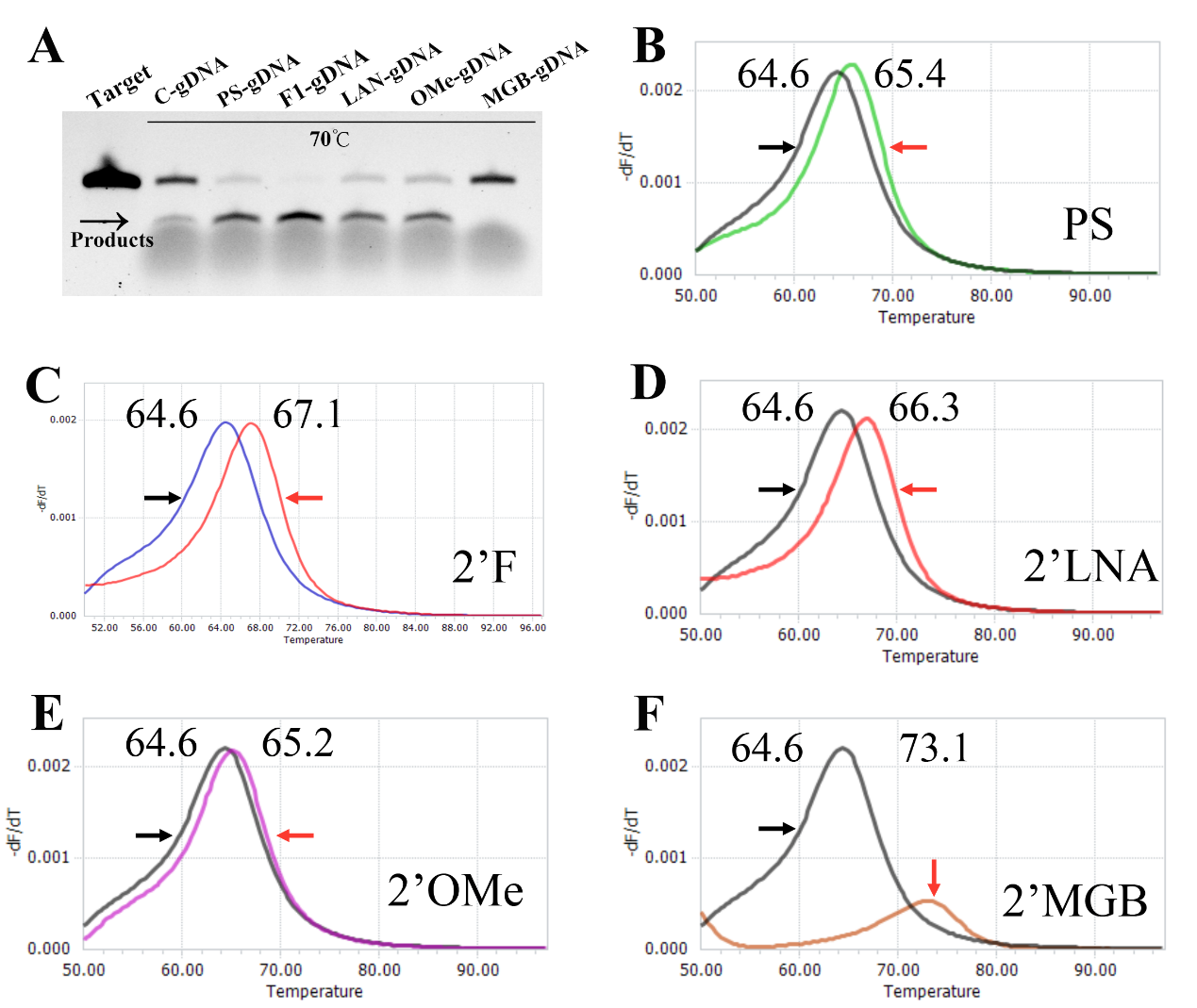


**Figure S4.** **Affection on TtAgo’s activity and gDNA’ Tm caused by chemical modifications.** (A) The affection on TtAgo’s dsDNA cleavage activity. (B-F) Tm comparison between canonical DNA and PS-, 2’F-, 2’OMe-, LNA- or MGB-gDNA. The black arrow indicates melting curve of canonical gDNA, and the red arrow indicates melting curve of chemically modified gDNA.


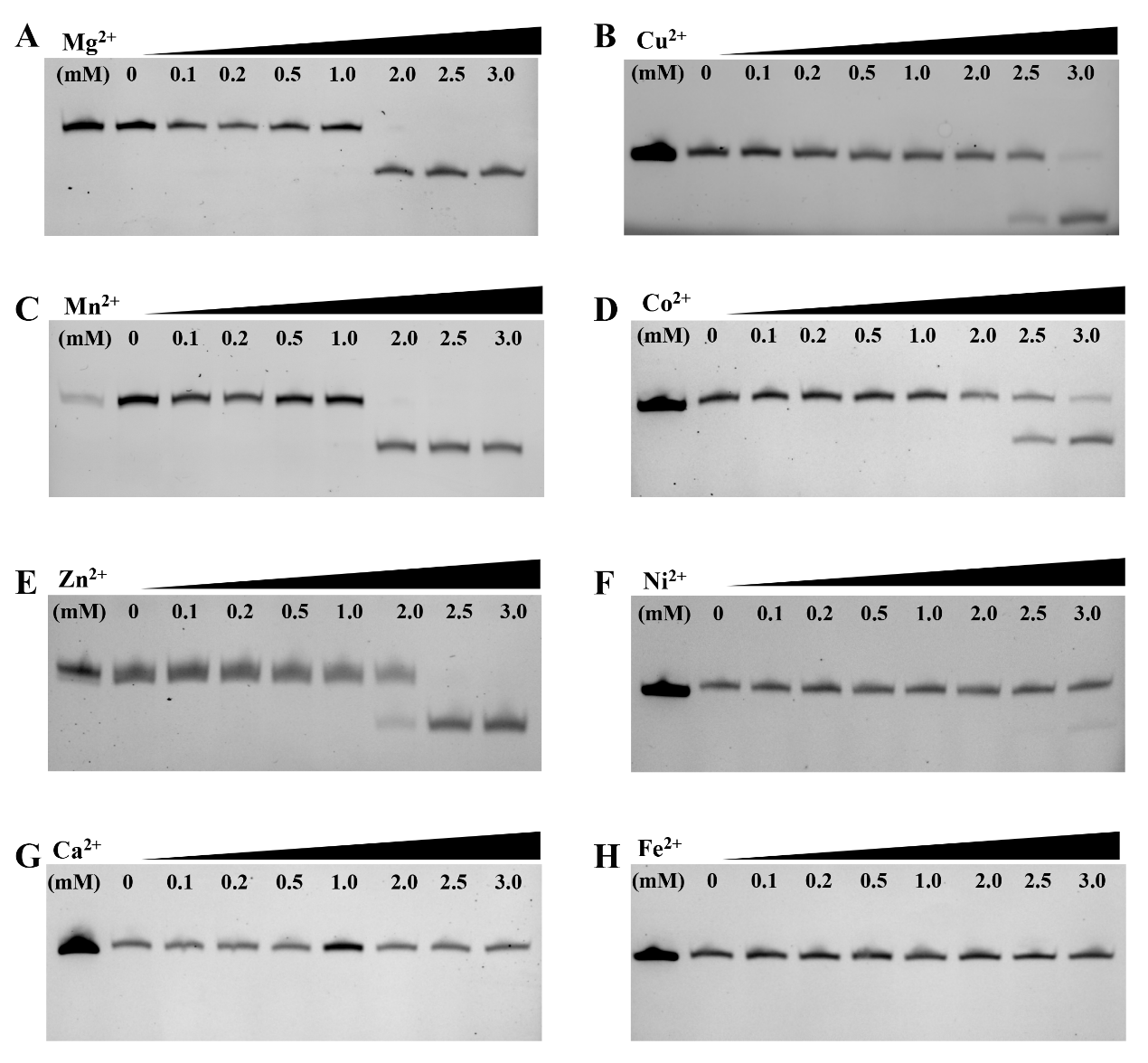


**Figure S5. The TtAgo activity affected by divalent metal ions.** The FATE was performed with FAM-labeled dsDNA target for at 70℃ for 30 min. Our results showed that the FATE need Mg^2+^, Cu^2+^, Mn^2+^, Co^2+^ or Zn^2+^ to function, not Ni^2+^, Ca^2+^and Fe^2+^.


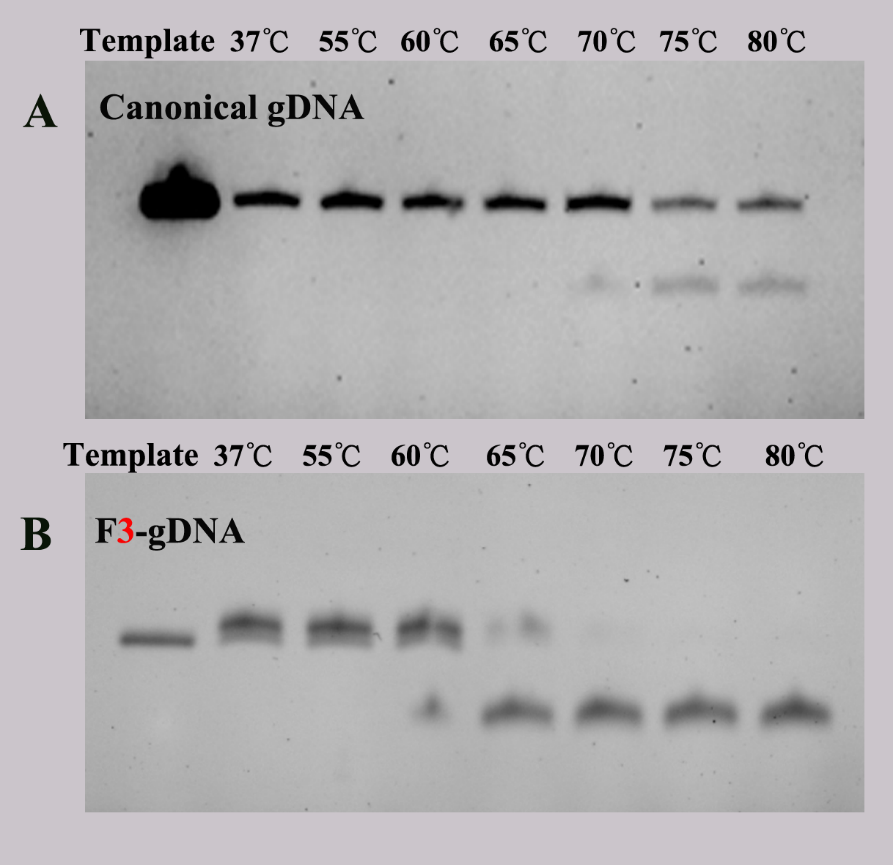


**Figure S6. Comparison the performance between canonical gDNA and F3-gDNA at different temperature.** (A) The canonical gDNA/TtAgo mediated dsDNA cleavage. (B) The FATE mediated dsDNA cleavage.


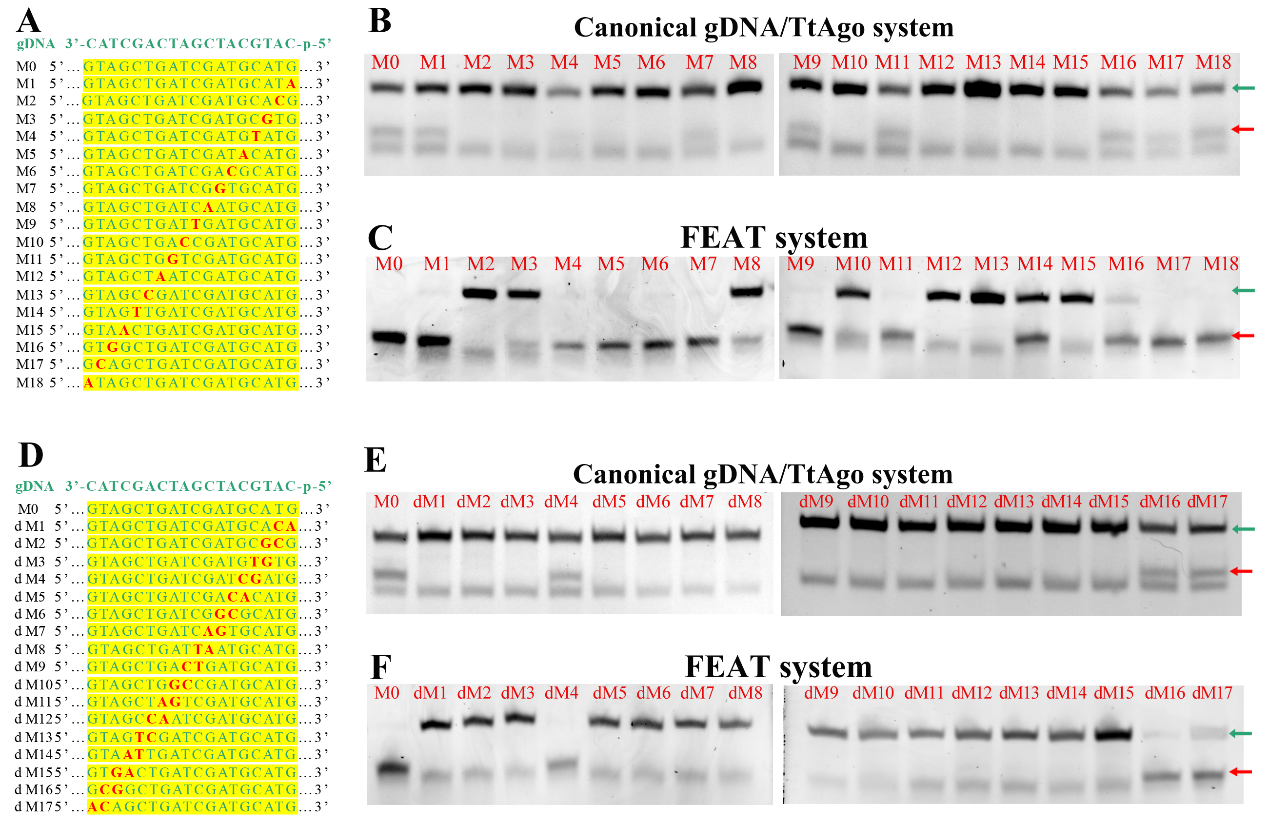


**Figure S7. Specificity comparison between** FATE and canonical gDNA/TtAgo. (A) The targets with single mismatch. (B) Single mismatch discrimination capacity of canonical gDNA/TtAgo. (C) Single mismatch discrimination capacity of FATE. (D) The targets with double-mismatched bases. (E) Double mismatch discrimination capacity of canonical gDNA/TtAgo. (F) Double mismatch discrimination capacity of FATE. The green arrow indicates target dsDNA, and the red arrow indicates cleavage product.


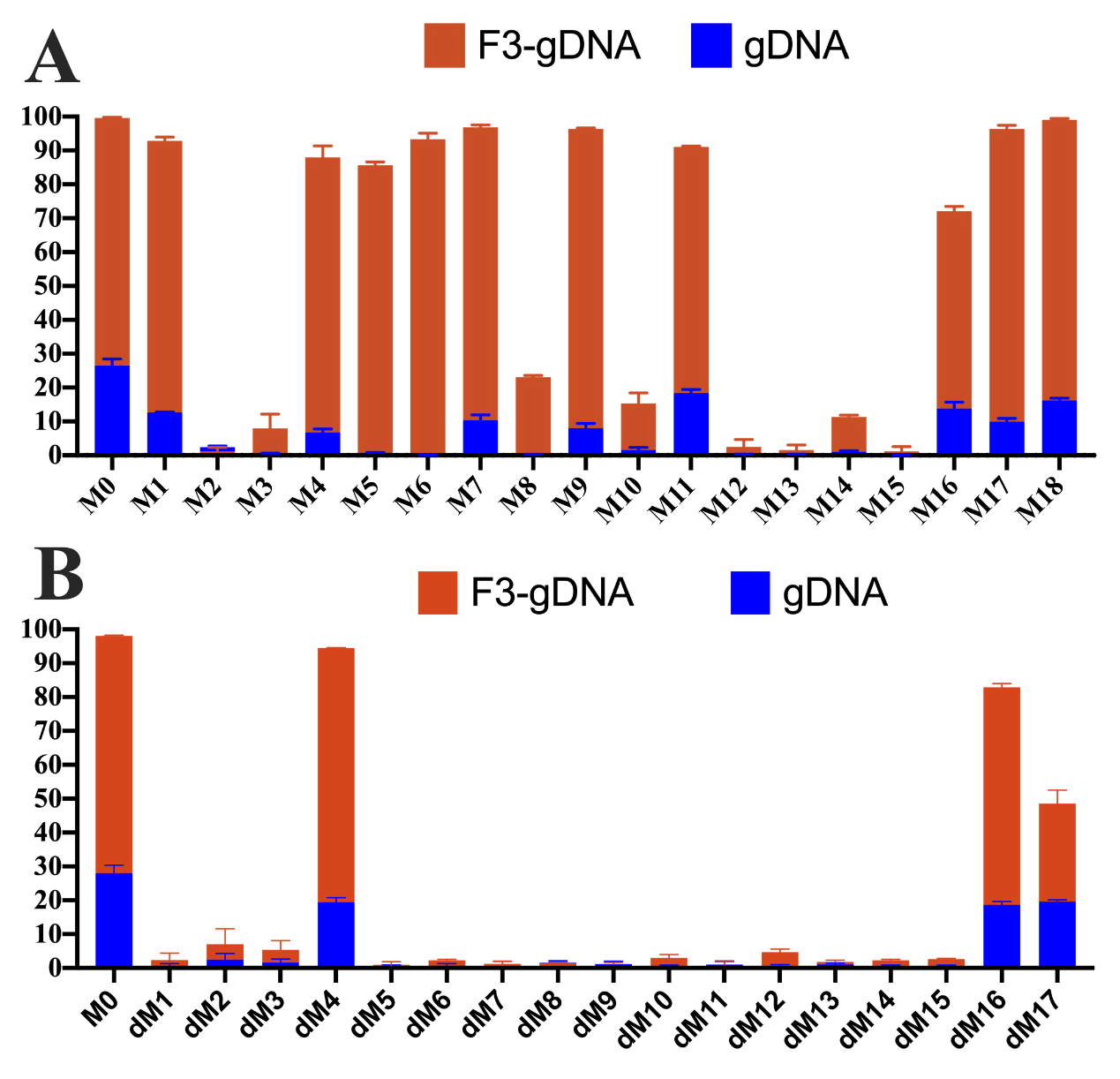


**Figure S8. Quantitative analysis of the cleavage activity (data from Figure S7).** The FATE system showed identical cleavage activity to the canonical gDNA/TtAgo at the discriminable mismatch sites, such as M2, M12, M13, M15, dM1, dM5, dM6, and so on. Except for these discriminable mismatch sites, the FATE system also showed significantly higher activity than the canonical gDNA/TtAgo system.


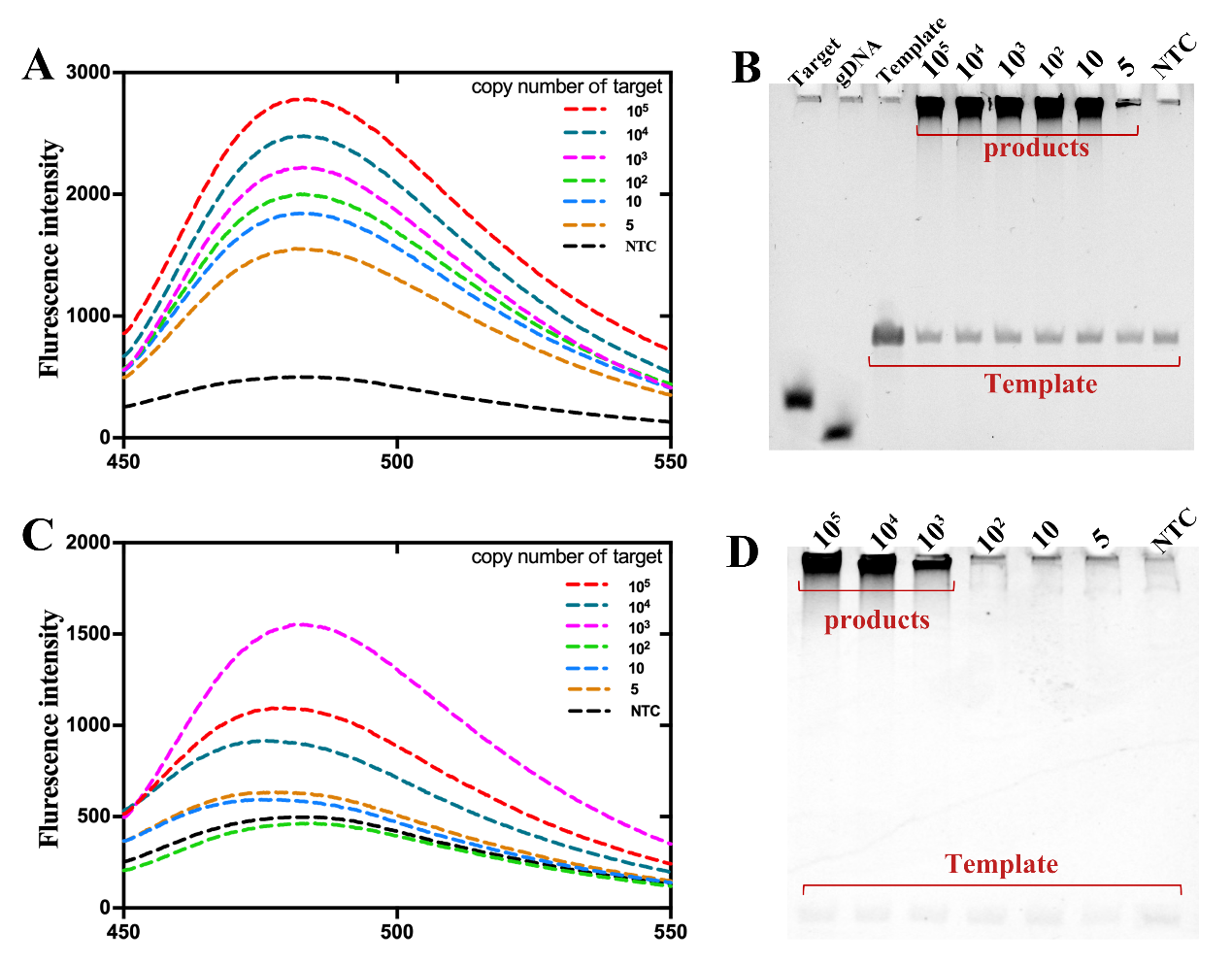


**Figure S9. Sensitivity of FAST and CAST.** (A) FAST assays were carried out by using 1 to 105 copies of chemically synthesized miR-21. (B) PAGE analysis the products of picture A. (C) CAST assays carried out by using 1 to 105 copies of chemically synthesized miR-21. (D) PAGE analysis the products of picture C.


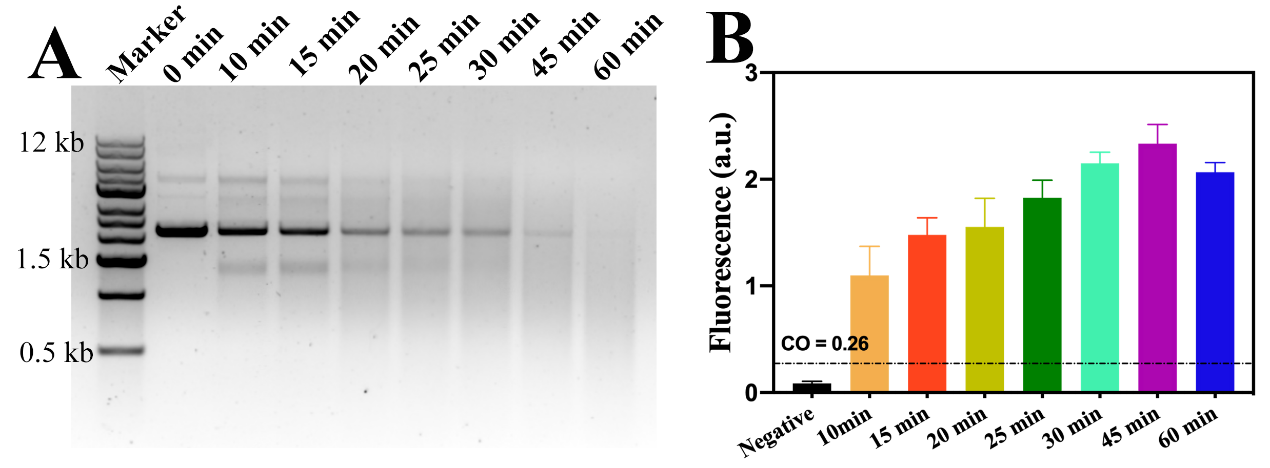


**Figure S10. FAST for dsDNA detection.** (A) Duplex-specific nuclease mediated dsDNA fragment. (B) The fragmented dsDNA analyzed by FAST. To expand the FAST application to dsDNA detection, duplex-specific nuclease (DSN) was employed for fragmenting dsDNA. A plasmid containing 1518 bp of Human papillomavirus L1 gene (pHPV-DNA) was fragmented by DSN at 37℃ for 0-60 min, consequentially generating FAST-specific fragments by chance. These fragments can be melted at the reaction temperature (65℃), therefore triggering FAST assay. The detecting performance increased with the fragmenting time, and reached maximum after 45min DSN digestion. On the basis of the successful dsDNA detection, we reasonably speculated that the FAST assay could be applied to ssDNA and mRNA detection by combining with proper sample pretreatment. Notably, the DSN mediated dsDNA fragment is random, so the reproducibility of FAST-dsDNA assay is unsatisfactory for sample with low concentration.
